# Soil and river water salinity dynamics in coastal Bangladesh

**DOI:** 10.1038/s41598-025-30639-5

**Published:** 2025-12-17

**Authors:** Ahmed Z. Rahman, Mohammad Shamsudduha, Md Izazul Haq, Md Hanif, Md Sanaul Islam, Syed S. Islam, Amarendranath Biswas, Richard G. Taylor

**Affiliations:** 1 Department of Risk and Disaster Reduction, University College London, London, UK; 2 Ministry of Public Administration, Bangladesh Secretariat, Dhaka, Bangladesh; 3 Department of Geography, University College London, London, UK; 4 Department of Disaster Science and Climate Resilience, University of Dhaka, Dhaka, Bangladesh; 5 Soil, Water and Environment Discipline, Khulna University, Khulna, Bangladesh; 6 Salinity Management and Research Centre, Soil Resource Development Institute, Khulna, Bangladesh

**Keywords:** Soil and water salinity, Seasonality, Cyclones, Climate adaptation, Bangladesh, Climate sciences, Environmental sciences, Hydrology

## Abstract

Changes in soil and water salinity pose critical challenges to agriculture, water management, and livelihoods in deltaic environments globally, and particularly in the densely populated Asian mega-deltas. Using observations from 24 stations over nearly two decades (2004–2022) in the Ganges-Brahmaputra-Meghna delta of Bangladesh, our scientific study examined the influences of seasonal weather and climate conditions, tropical cyclones, and hydrology on the seasonal variability in soil and river water salinity. We applied statistical analyses including cross-correlation, seasonal-trend decomposition, and wavelet analysis to explore the spatiotemporal dynamics of soil and river water salinity. We developed statistical models to assess how hydrological, meteorological, and climatic factors explain the variability of salinity. Pronounced seasonal fluctuations in soil and river water salinity are observed, with levels rising during the dry season and declining sharply during the monsoon season (June‒October). We also observed how tropical cyclones contribute to short-term spikes in both soil and river water salinity, with stronger impacts observed for those making a landfall during early monsoon period (April‒May). Storm-surge-driven polder breaches further exacerbate salinisation, compounded by land subsidence and rising sea levels. Statistical models reveal a significant positive association between soil and river water salinity and sea-surface salinity during the pre-to-early monsoon season. In contrast, the seasonal rise in sea levels during the monsoon coincides with reduced soil and river water salinity due to monsoon rainfall and freshwater discharges to the sea.

**Supplementary Information:**

The online version contains supplementary material available at 10.1038/s41598-025-30639-5.

## Introduction

**Fig. 1:**
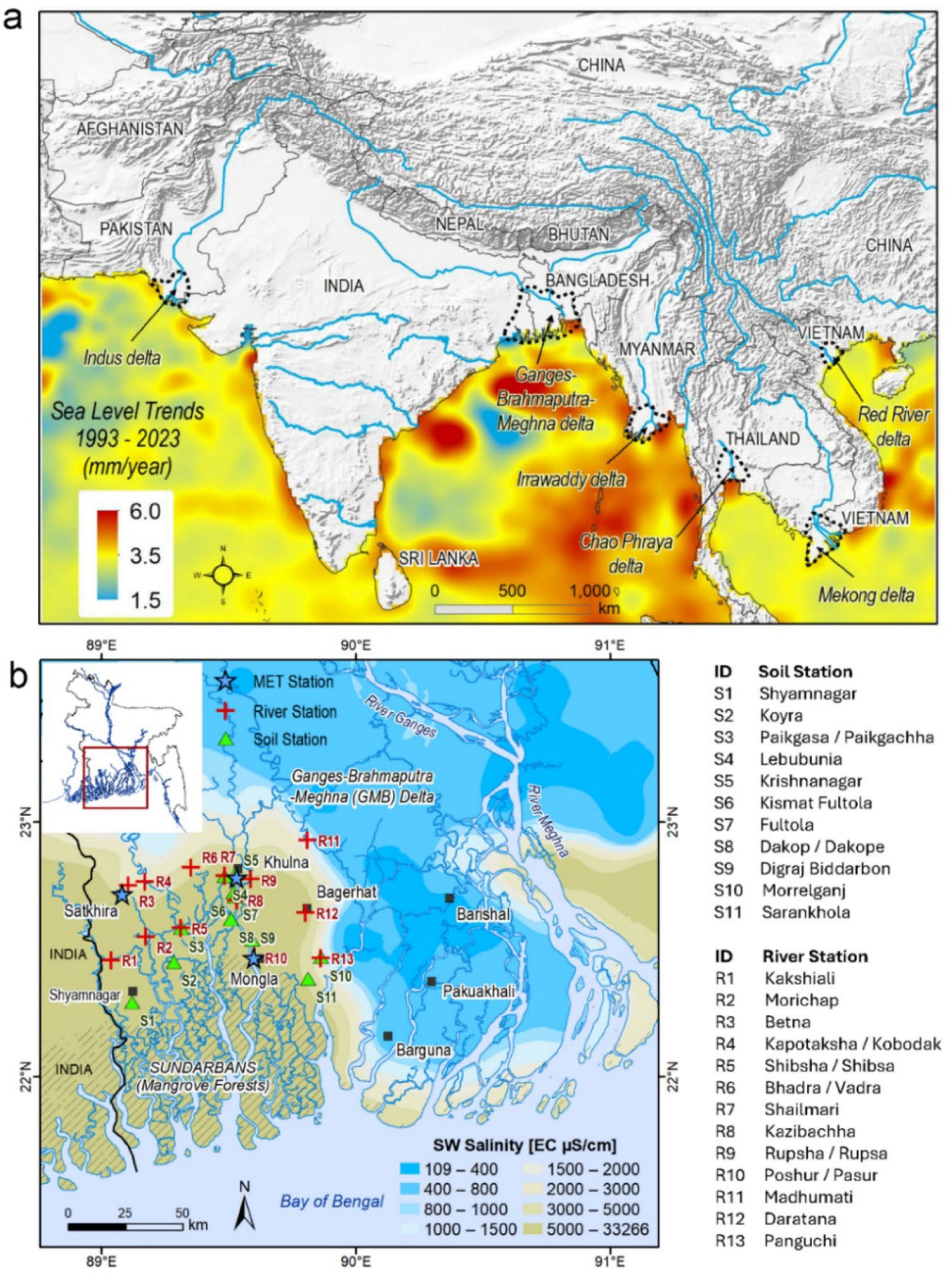
The locations of Asian mega-deltas and a high-resolution map of the Ganges-Brahmaputra-Meghna delta in Bangladesh. (**a**) Trends in sea levels (1993–2023) and the location of Asian mega-deltas (data source: Monthly sea level anomaly data at 0.25°×0.25° resolution from https://data.marine.copernicus.eu/products). (**b**) Mean surface water salinity map of the southern coastal Bangladesh (data source:^11^) and the location of soil and river water salinity monitoring stations. Data were processed and analysed in R GUI (R version 4.4.2). Maps were created using ArcGIS Desktop (v10.8).

In low-lying deltaic environments around the world, soil and water salinity pose significant challenges to food security, public health, and environmental sustainability^1,2^. Increased salinisation of soil, surface water, and coastal groundwater has become a critical problem, affecting agricultural lands, food production, and the livelihoods of millions of farmers in deltaic environments including the densely populated Asian mega-deltas. High soil salinity and its associated adverse effects on the environment, ecosystems, food security, and livelihoods have been observed in the Asian mega-deltas (Fig. 1a), including India^3^, the Indus River Delta of Pakistan^4^, the Mekong Delta of Vietnam^5^, the Yellow River Delta of China^6^, and the Ganges-Brahmaputra-Meghna Delta of Bangladesh^7^ and West Bengal, India^8^.

In Bangladesh, soil and surface water (i.e., river and pond waters) salinity is a growing concern, particularly in the coastal region^9^. Coastal Bangladesh consists of 19 districts (out of 64 districts) covering 30% of the country’s land area and is home to nearly 44 million people (BBS, 2022). Around 9 million of these people in the five southwestern coastal districts (Bagerhat, Barguna, Khulna, Pirojpur and Satkhira) are acutely affected by high water and soil salinity. Surface water (i.e., river water) in southwestern Bangladesh is mostly saline – an electrical conductivity of > 5000 µS/cm (Fig. 1b;^11^) with substantial seasonal variability. Several factors exacerbate soil and surface water salinity, including reduced freshwater flows from the upstream rivers^10^, geomorphological changes, storm surges, land subsidence^11^, irrigation, brackish-water shrimp farming^12,13^, and the construction of polders^14^. It is argued that climate change further intensifies the salinity problem through rising sea levels, increased frequency and intensity of tropical cyclones, and amplification of rainfall extremes^66^. However, robust evidence linking these climate-related changes to increased salinisation of soil and surface water remains limited.

Previous studies have explored the spatiotemporal dynamics of soil or river water salinity in coastal Bangladesh. Surface water in this study primarily refers to river water, and the two terms are used interchangeably. Past studies have explored water and soil salinity primarily through visual or statistical means^9,10,15^. Kawser et al. ^16^ investigated changes in soil salinity in southeastern Noakhali coastal region of Bangladesh using data from two survey years: 1996 and 2012. Rahman and Rahman^17^and Jahan et al^18^ focused on water salinity in southwestern region with limited observational data. Our literature review reveals that globally, there is a lack of long-term monitoring data on soil salinity. Due to this dearth of ground-based observations, Earth Observation data have been applied to explore changes in soil salinity. For example, satellite data (e.g., Landsat and Sentinel) were used to characterise soil salinity in Hungary^19^, soil salinity trends in the Bakhtegan Salt Lake region of Iran^20^, the impact of drainage network on soil salinity in Northeast Iran ^21^, and soil salinity dynamics in Kuwait^22^. Sarkar, et al^23^ calculated soil salinity indicators using Landsat satellite images and machine learning techniques to map soil salinity in one district in southwestern coastal Bangladesh. Bhuyan, et al^15^ collected both water and soil samples on a monthly frequency (November 2020 and June 2021) as well as a one-off 562 soil samples in April 2021 from four coastal districts (Barishal, Borguna, Jalakhati, Patuakhali) in south-central Bangladesh. They also calculated salinity index from Landsat satellite data and concluded that soil and water salinity gradually increased from January onwards and peaked during May.

Despite recent advancements in remote sensing, machine learning, and electromagnetic methods for soil salinity monitoring^24^, there remains limited integration of locally relevant remote-sensing and geophysical datasets and field-level time-series monitoring data to assess soil salinity dynamics and understand the complex interplay of factors contributing to soil and water salinity in coastal Bangladesh and other Asian mega-deltas. Research to date examines salinity in isolation, focusing on either soil salinity, surface water salinity, or groundwater salinity, without addressing the interconnected influences of hydrological, meteorological, climatic, and anthropogenic factors^9–11,15^. No previous study in Bangladesh has simultaneously considered the roles of hydrological factors (e.g., river discharge, surface water levels), meteorological influences (e.g., tropical cyclones, rainfall, and temperature patterns), climate change impacts (e.g., sea level rise, long-term rainfall distribution, and temperature increases), and anthropogenic activities (e.g., polder construction, land-use changes, and agricultural practices) in driving/controlling changes in soil and water salinity. A key barrier to understanding these dynamics has been the lack of long-term monitoring data.

The novelty of this study lies in its presentation of rare time series of combined monthly monitoring of soil and river water salinity across three coastal districts in Bangladesh, covering the period from January 2004 to June 2022. We analyse these salinity data both visually by plotting and observing time-series data and statistically, examining their relationship with hydrological, meteorological, climatic, and anthropogenic factors that influence the spatiotemporal dynamics of river water and soil salinity. Our analysis addresses the following key questions: (1) What drives the seasonal variation in soil and river water salinity? (2) How do tropical cyclones and local weather influence soil and river water salinity? and (3) What are the impacts of rising sea levels, sea salinity, terrestrial hydrology, and climate variability on soil and river water salinity?

In Bangladesh and other Asian mega-deltas, water and soil salinity are major concerns, and both infrastructure development and climate adaptation efforts are underway to address the adverse impacts of salinisation. Understanding the mechanisms driving soil and water salinity is essential for informing effective climate adaptation and resilience-building efforts. The findings from this research seek to contribute to better-targeted and more effective climate adaptation efforts in salinity-affected regions.

## Results

### Pronounced seasonal variability in soil and river water salinity

**Fig. 2:**
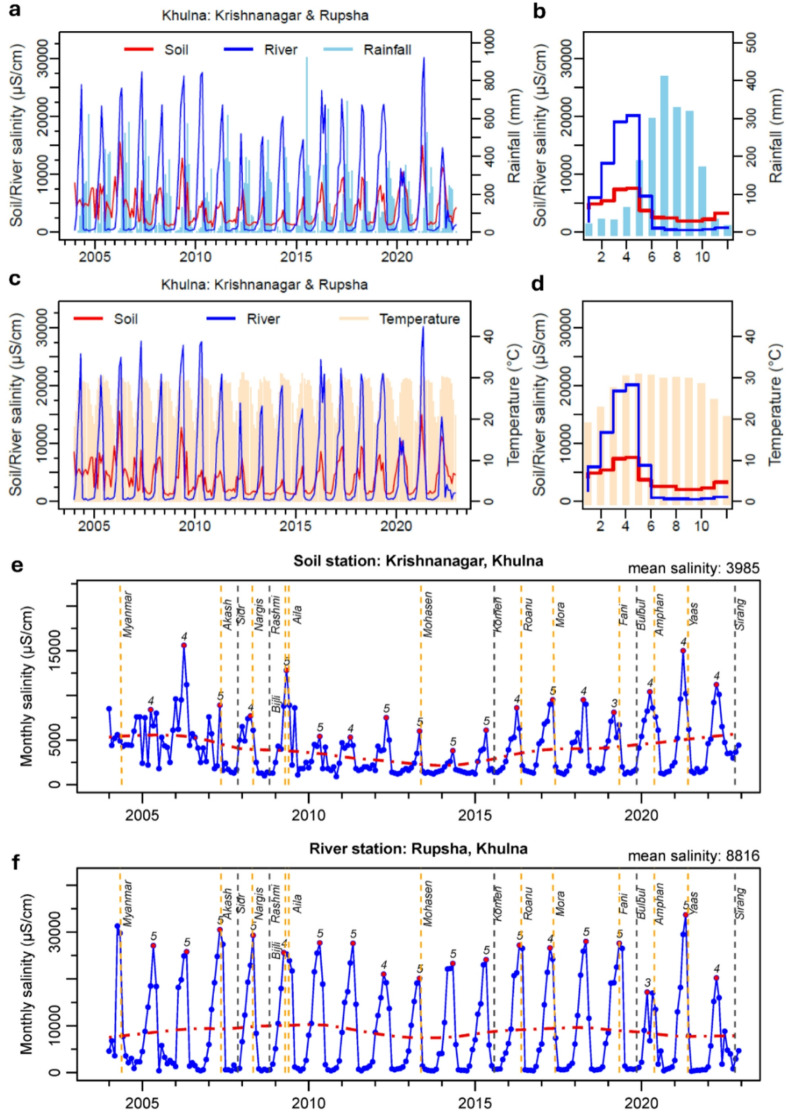
Time-series plots of soil and river water salinity data in Bangladesh. (**a**) Soil and river water salinity and rainfall at Krishnanagar and Rupsha (**b**) Mean monthly salinity and rainfall (**c**) Soil and river water salinity and temperature (**d**) Mean monthly salinity and temperature (**e**) Soil salinity at Krishnanagar plotted with the timing of tropical cyclones shown as vertical dash lines; the month of peak seasonal salinity in each year is marked with a red solid circle. All time-series datasets were processed, analysed and visualised in R GUI (R version 4.4.2).

We observe that soil and surface water (i.e., river channels) salinity (measured in terms of Electrical Conductivity or EC) values are highly seasonal in nature. Pronounced seasonal fluctuations occur between dry, summer (March to May) and wet, monsoon (June to October) seasons in which average minimum and maximum values of soil salinity (*n* = 11) range from ~ 1,000 to 22,100 µS/cm and river water salinity (*n* = 13) range from ~ 370 to 30,400 µS/cm. At Krishnanagar station (S5) in Khulna (Figs. 2b and 2), soil salinity rises slowly from a monthly mean EC value of 1,600 µS/cm in September to 7,600 µS/cm in May. Similarly, monthly mean surface water salinity in River Rupsha station (R9) rises slowly from 300 µS/cm to a value of 20,000 µS/cm in the month of May. Similar time series of soil and surface water salinity data for other monitoring stations are presented in the supplementary information (Figure S1).

The mean Pearson and Spearman correlation coefficients between soil and river water salinity time-series (*n* = 13 × 11 = 143 pairs) data are 0.67 and 0.71. Furthermore, the Cross Correlation Function (CCF) analysis shows correlation with a lag in the monthly time-series data in the soil and river water salinity time-series records (January 2004 to June 2022). CCF results (see Table S1) show a mean correlation of 0.72 with a standard deviation of 0.11 with zero-month lag for 100 out of 143 pairs of stations; with a 1-month lag, the mean cross correlation is 0.63 with a standard deviation of 0.14 for 36 pairs of stations. However, lags of less than a month may exist in many of these paired soil and river water salinity stations, yet we are unable to confirm this due to the lack of high frequency (i.e., hourly or daily) salinity observations. To explore the seasonal component in the time-series data, we applied the Seasonal and Trend decomposition using Loess (STL) method^25^. STL decomposition reveals (Figure S4) that the seasonal component represents nearly 60% and 78% of the variability observed in the soil and river water salinity time-series, respectively. Wavelet analysis (Figure S5) reveals periodicity in the salinity data as it decomposes the time series into components associated with different time scales or frequencies^26^. Both soil and river water salinity time-series records clearly show annual seasonality (12-month cycle) though a 6-month cycle is visible in some stations. Wavelet coherence (Figures S6 and S7) analysis also shows correlations between soil and river water salinity as well as with monthly rainfall data from the Bangladesh Meteorological Department (BMD).

**Fig. 3:**
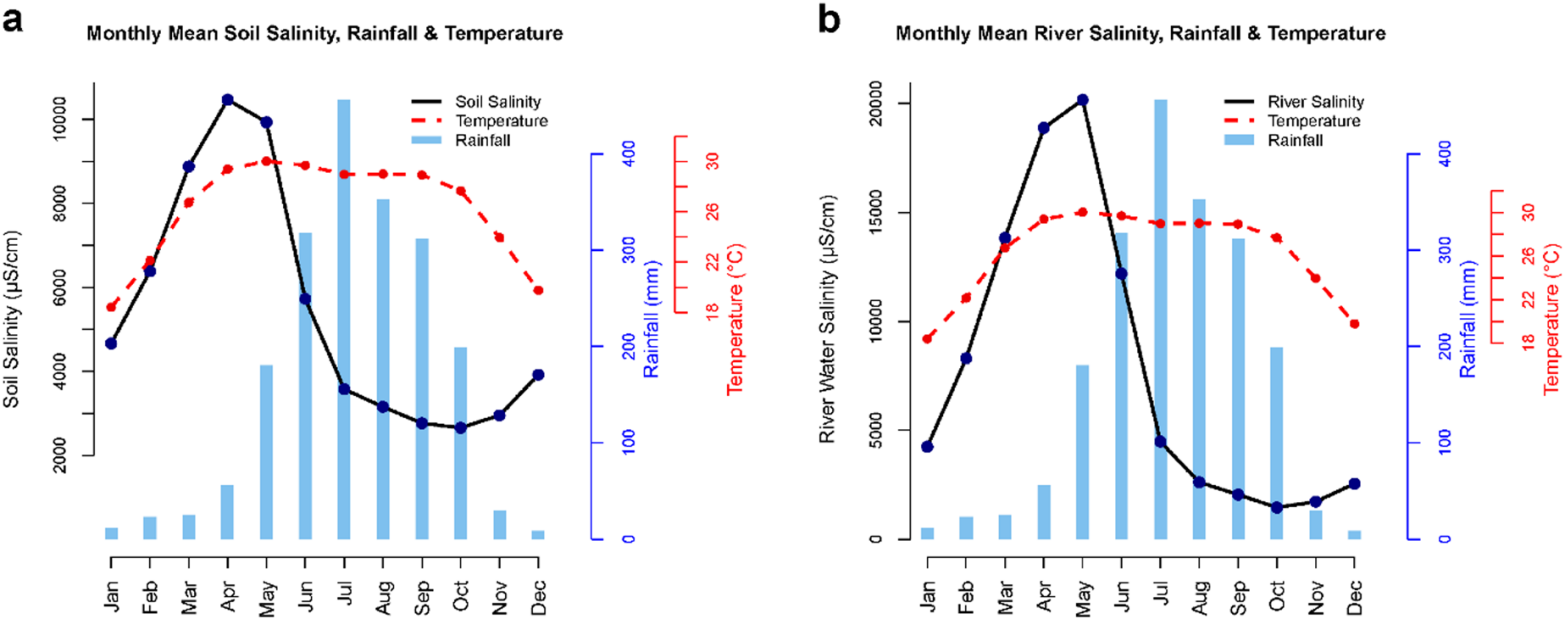
Monthly seasonality plots of soil and river water salinity with monthly means of rainfall and temperature. (**a**) Soil salinity (**b**) Water salinity. Mean monthly rainfall and temperature from Khulna, Mongla, Patuakhali and Satkhira are also shown on the plots. All time-series datasets were processed, analysed and visualised in R GUI (R version 4.4.2).

**Fig. 4:**
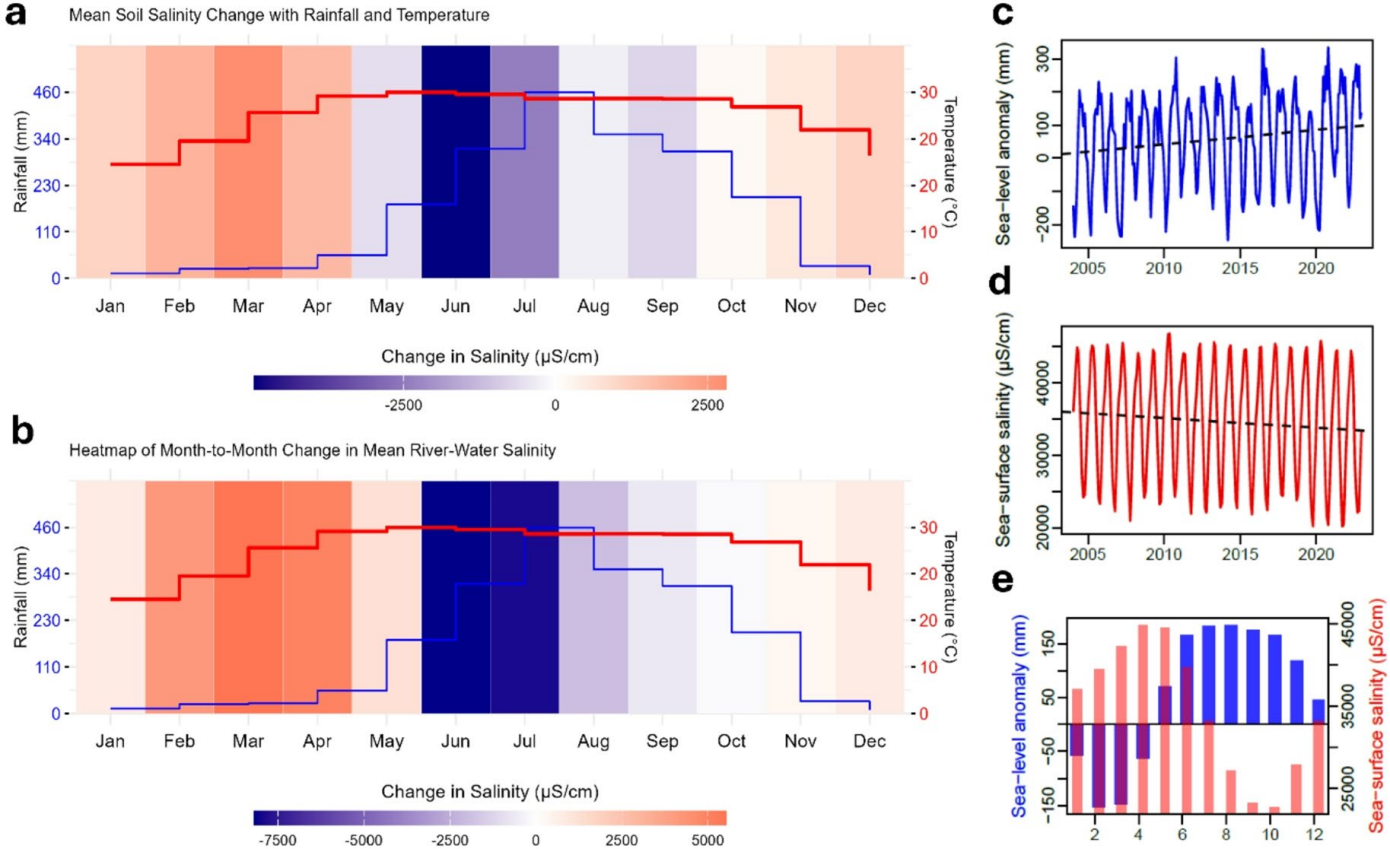
Time-series plots of soil and river water salinity data in Bangladesh. (**a**) Mean monthly soil salinity change with rainfall and temperature at Krishnanagar. (**b**) Same as (**a**) but for river water salinity. (**c**) Monthly time-series of sea-level anomaly in the Bay of Bengal. (**d**) Monthly time-series of sea surface salinity in the Bay of Bengal. (**e**) Mean monthly seasonality in sea-level anomaly and sea-surface salinity. All time-series datasets were processed, analysed and visualised in R GUI (R version 4.4.2).

### Effects of seasonal weather and climate on seasonal variability of salinity

Local temperature and rainfall have significant effects on the seasonal variability in the soil and river water salinity observed in all 24 monthly time-series records from southwestern Bangladesh. Visually, the monthly climatology plots of rainfall and temperature with salinity show dynamic and non-linear associations (Fig. 2). Rises in monthly soil and river water salinity closely follow the rise in monthly temperature from January onward that peaks around April-May until the monsoon rainfall begins. Seasonal decreases in soil and river water salinity from their peak levels then closely follow the monsoon rainfall. The effect of temperature and evaporation on both soil and river water salinity during the monsoon season is minimal and largely negated by the monsoon rainfall from June to October (Fig. 3). The lowest levels of salinity are observed in October, following the bulk of the seasonal rainfall from June to September. The rate of change in salinity varies substantially between the two consecutive months with the highest change (i.e., decrease in salinity) observed in June and July (Fig. 4a and b). Both soil and river water salinity begin to rise upward from October while both rainfall and temperature continue to decline resulting in an inverse association. Interestingly, this coincides with the rising seasonal sea surface salinity from the beginning of November (Fig. 3e).

In addition, cross correlation using CCF during the dry 6-month period (November to April) between mean soil salinity and temperature is 0.66 with and between river water salinity and temperature is 0.79 at zero lag indicating no delayed temperature effect. Similarly, a cross correlation during the wet 6-month period (May to October) between mean soil salinity and rainfall is 0.60 and between river water salinity and rainfall is 0.55 with a 2-month time lag indicating a delayed effect of flushing and dilution of salinity during the monsoon season. Soil salinity starts to drop early in the monsoon season with the first spell of rains in May, (Figs. 2a and 3a) whereas river water salinity does not begin to drop until June as there is a delay between rainfall and river discharge, especially from the upstream regions.

### Long-term patterns in soil and water salinity

We applied linear trend and non-parametric Sen’s slope^27^, as well as STL decomposition^25^ methods to characterise long-term patterns in soil and river water salinity data (Figs. 2 and S4); wavelet analysis shows periodicity and coherence in records (Figures S5  to S7). Overall, long-term patterns (Jan 2004 to Jun 2022) in both soil and river water salinity show mean decreasing trends: −176 µS/cm/year in soil salinity stations (*n* = 11) and − 93 µS/cm/year in river water salinity stations (*n* = 13). Sen’s slopes are − 129 µS/cm/year and − 35 µS/cm/year respectively. Seasonal Sen’s slopes are also negative (Tables S2 and S3).

Interestingly, we observe that the trend in soil salinity at some sites such as Krishnanagar (Khulna) is not monotonic: it shows a declining pattern in the first part of the time series (2004–2014) and a rising pattern in the latter part (2014–2022) that ultimately result in no consistent overall trend over the entire time series (Fig. 2e). Critically, dry-season soil salinity levels have been steadily increasing since 2014. The long-term trend in river water salinity at Rupsha River (Khulna) shows (Fig. 2f) a similar decreasing pattern though the magnitude of trend is smaller compared to soil salinity (Table S2). Application of the Seasonal-Trend decomposition using LOESS (STL) on Krishnanagar soil salinity time-series data (Figure S4) reveals a very small trend component representing around 15% variability of the total variance observed in the time-series data. Seasonal and irregular or residual components represent 47% and 37% variability respectively at Krishnanagar site (Table S2). On average, the trend, seasonality and irregular components represent 15%, 60% and 25% variability respectively in all soil salinity (*n* = 11) time-series data (Table S2). In comparison, on average, the trend, seasonality and irregular components represent 3%, 78% and 19% variability respectively in all river water salinity (*n* = 13) time-series data (Table S3). These analyses clearly demonstrate strong seasonal variations in soil and river water salinity in southwest Bangladesh.

### Drivers of soil and river water salinity: insights from statistical modelling

To explain seasonal and long-term variability in soil and river water salinity data, we applied Multiple Linear Regression (MLR) and Generalised Linear Models (GLMs) through mean monthly as well as mean annual time-series records of 11 soil and 13 river water salinity stations. Twelve covariate datasets (meteorology: rainfall, temperature and cyclone; hydrology: evapotranspiration, surface runoff, soil moisture, surface water levels, river discharge; climatology: sea level anomaly and sea surface salinity; and anthropogenic factors: groundwater levels and Normalised Difference Vegetation Index or NDVI) are considered in the models to explain the variability observed in the salinity time-series data. The use of NDVI as a variable in the models represents vegetation health and its presence and absence seasonally. NDVI captures seasonal vegetation dynamics that can influence salinity patterns and shows an inverse association with salinity^28^. For example, dense vegetation can reduce evaporation and enhance infiltration, potentially moderating surface salinity buildup. Conversely, bare or degraded land, often associated with high salinity, shows low NDVI values. A series of MLR models is developed to explain the seasonal and annual mean variability observed in soil and river water salinity data. One of the main challenges we encounter is that covariates are highly seasonal (Figures S8) as well as highly correlated to each other (Figure S9). When predictors are highly correlated, multicollinearity can distort the interpretation of model coefficients and the direction of association. Further details on the exploratory analyses and statistical models are provided in the methods section.

Twelve covariates or predictors used in these models are: meteorological (AvgRain = average rainfall (mm), AvgTemp = average temperature (°C), AvgET = average evapotranspiration (mm) and Cyclone = occurrence of tropical cyclones as binary variable); climatological (SLanom = sea level anomaly (m); Ssalinity = sea surface salinity (µS/cm)); hydrological and hydrogeological (AvgRO = surface runoff (mm), AvgSM = average soil moisture (mm), RivDisch = river discharge (m^3^/sec), SWLm = surface water levels (m), GWLbgl = groundwater level as depth below ground level (m, bgl)); and land-use/land-cover or anthropogenic factors (i.e., NDVI = Normalised Difference Vegetation Index and groundwater levels).

Results from MLR models reveal interesting associations between soil salinity and predictor variables, as well as between river water salinity and the predictor variables (Table 1). A series of models were created using various factors to test the explanatory power of certain group of variables on soil and river water salinity (Tables S4 to S9). In a log-linear model, a one-unit increase in an independent variable means a multiplicative change in the response variable (i.e., salinity). Since we use the natural logarithm (Figure S10) of soil and river water salinity data in the model, the interpretation of the model coefficients is that a one-unit increase in a predictor variable is associated with a change (depends on the sign) in the logarithm of salinity by the corresponding model coefficient of that predictor or explanatory variable. For example, a statistically significant (*p* value < 0.001) positive association is modelled between temperature and soil salinity. This model result (temperature, *β* = 0.0617) suggests that a one-unit increase in temperature (seasonal or long-term change) leads to a percentage change (i.e., increase) in soil salinity, $\:y={(e}^{0.0617}-1)\:x\:100\%\approx\:6.4\%$ (Table 1). Rainfall shows a strong correlation with surface runoff so that model results are affected by the multicollinearity effect. So, in the model we disregard surface runoff. NDVI, which is a measure of vegetation health (i.e., higher values typically indicate healthy, dense vegetation, and lower values indicate less healthy or sparse vegetation), has a negative association with soil salinity (*β*=−1.8144, *p* value < 0.001). Since NDVI values range only from − 1 to + 1 and typically change incrementally, this model result suggests that a 10% increase in NDVI is associated with an approximate 8.5% decrease in soil salinity. Groundwater levels, expressed as negative values representing depth below ground level (m bgl) has a potentially significant positive association (*β* = 0.2091, *p* value = 0.058) with soil salinity suggesting that an increase (less negative values) or shallowing of groundwater levels by 1 m would lead to an increase in soil salinity by 23%. The model also suggests that the occurrence of tropical cyclones has a positive (*β* = 0.24994, *p* value = 0.023) influence on soil salinity (i.e., soil salinity can potentially increase by 28% per additional cyclone occurrence). Sea surface salinity has a positive statistically significant association (*β* = 0.00005, *p* value < 0.001) with soil salinity whereas sea-level anomaly has a negative (*β*=−0.00179, *p* value < 0.001) statistically significant associations with soil salinity. Model results are similar for the river water salinity data and model predictors (Table 1) with an exception for groundwater level which shows a statistically significant negative association (*β*=−0.3543, *p* value = 0.001) with river water salinity suggesting that shallowing of groundwater levels by 1 m would lead to a decrease in river water salinity by 30%. Overall, the fitted MLR models adequately explain > 70% (*R*^*2*^ > 0.70, *p* value < 0.001 for soil salinity) and > 90% (*R*^*2*^ ≥ 0.90, *p* value < 0.001 for river water salinity) variability observed in soil and river water salinity data.

In addition to MLRs, we apply Generalised Linear Models (GLMs) to explain the variability observed in the mean soil and mean river water salinity time-series data. GLM results are similar to MLR models. GLMs also adequately capture 73% and 90% variability observed in the soil salinity and river water salinity time-series datasets. To assess the contribution of individual covariates to model performance, stepAIC() was applied in R for stepwise selection, which identifies a subset of predictors that minimise the Akaike Information Criterion (AIC), balancing model complexity and goodness of fit.

Model fitting was assessed through the standard model diagnostic plots (Figures S11 and S12). Model performance metrics such as *R*², adjusted *R*², AIC, and log-likelihood provide insights into model fit, but direct comparison is not valid here because the models were fitted on datasets with different sample sizes resulting from missing values versus imputed data. Although the full dataset contains 222 rows (months), the first model was fit to a time-aligned subset of 117 rows, of which 40 were excluded due to missing values in the model variables, resulting in 77 complete observations used for fitting. The second model was fitted with all 222 rows as missing values for the response variable and covariates were imputed using the Random Forest algorithm from the missForest R package – a standard practice in statistical modelling of environmental datasets where observations are often missing or even reported below detection limits^29^.

**Table 1:** Summary statistics of multiple linear regression (MLR) models for soil and river water salinity in Southwestern coastal Bangladesh.

	log(soil salinity)		log(soil salinity)		log(river salinity)		log(river salinity)	
*Predictors (explanatory variables)*	*Estimates*	*P*	*Estimates*	*p*	*Estimates*	*p*	*Estimates*	*p*
(Intercept)	6.7626^***^	< 0.001	6.0927^***^	< 0.001	2.689	0.057	4.0512^***^	< 0.001
Avgrain	−0.0003	0.800	−0.00034	0.158	−0.0004	0.666	0.0004	0.064
AvgET	−0.0092	0.078	-	-	−0.0073	0.126	-	-
Avgtemp	0.1316^***^	< 0.001	0.0617^***^	< 0.001	0.1081^**^	0.001	0.0608^***^	< 0.001
AvgSM	−0.0002	0.947	-	-	0.0012	0.607	-	-
AvgRO	−0.0006	0.880	-	-	−0.0028	0.473	-	-
SLanom	−0.0015^**^	0.008	−0.0018^***^	< 0.001	0.0003	0.537	−0.0006^*^	0.037
Ssalinity	0.00001	0.576	0.00005^***^	< 0.001	0.00008^***^	< 0.001	0.00007^***^	< 0.001
Rivdisch	−0.00004	0.252	−0.00007	0.091	−0.00000	0.935	−0.00009^*^	0.018
Cyclone	0.17960	0.523	0.2499^*^	0.023	0.1888	0.467	0.0354	0.730
SWLm	0.8900^***^	< 0.001	0.5022^***^	0.001	−0.0693	0.699	−0.1452	0.315
GWLbgl	0.2331	0.208	0.2091	0.058	−0.25255	0.140	−0.3543^***^	0.001
NDVI	−2.9647^***^	< 0.001	−1.8144^***^	0.001	−0.0918	0.890	−0.2988	0.555
RivDisch × SWLm	-	-	0.00003	0.181	-	-	0.00005^*^	0.034
Observations		77		222		77		222
R^2^/R^2^ adjusted		0.78/0.73		0.71/0.70		0.92/0.91		0.89/0.89
Model *p* value		< 0.001		< 0.001		< 0.001		< 0.001
AIC (akaike information criterion)		1398.49		3961.35		1365.79		3932.91
log-likelihood		−19.33		−91.76		−13.09		−76.98

*p < 0.05 ** *p* < 0.01 *** *p* < 0.001.

**Table 2:** Summary statistics of generalised linear models (GLMs) for soil and river water salinity in Southwestern coastal Bangladesh.

	log(soil salinity)		log(river salinity)	
*Predictors (explanatory variables)*	*Estimates*	*p*	*Estimates*	*p*
(Intercept)	6.1116^***^	< 0.001	4.29082 ^***^	< 0.001
Avgrain	−0.0003	0.204	0.00064 ^**^	0.004
Avgtemp	0.0643^***^	< 0.001	0.05796 ^***^	< 0.001
SLanom	−0.0017^***^	< 0.001	−0.00053	0.060
Ssalinity	0.00005^***^	< 0.001	0.00007 ^***^	< 0.001
Rivdisch	−0.00009^*^	0.028	−0.00010 ^**^	0.007
SWLm	0.5552^***^	< 0.001	−0.14546	0.300
Cyclone	0.2290^*^	0.031	−0.01731	0.862
GWLbgl	0.2434^*^	0.023	−0.37974 ^***^	< 0.001
NDVI	−1.9184^***^	< 0.001	−0.45940	0.350
RivDisch × SWLm (interaction term)	0.00004	0.072	0.00005 ^*^	0.017
Observations	222		222	
AIC (akaike information criterion)	3959.70		3927.13	
Pseudo R^2^ (Nagelkerke)	0.73		0.90	
log-likelihood	−1967.85		−1951.56	

* *p* < 0.05 ** *p* < 0.01 *** *p* < 0.001

## Discussion

Here, we discuss the observed seasonal, annual and decadal-scale dynamics of soil and water salinity and influences of hydrological, climatological and anthropogenic drivers in the southwestern coastal region of Bangladesh. One of the key features of our analyses is that it reveals substantial seasonal variations (i.e., up to 2 orders of magnitude) in monthly soil and river water salinity. This outcome is consistent with the findings from previous, localised studies of soil salinity^9,16^ and regional-scale analyses of surface water salinity in coastal Bangladesh^10,11^. The novelty in our study lies in the detailed characterisation of soil and river water salinity from observations and their statistical associations with the local weather (i.e., temperature and rainfall) and climate change (i.e., rising sea levels).

Seasonal soil and surface water salinity rises steadily as temperature increases from winter to summer months and then falls very quickly (i.e., within a couple of months) as soon as the monsoon season begins. This association clearly demonstrates the critical role of local weather in the seasonal dynamics of soil and river water salinity in coastal Bangladesh. Exploratory analyses and visualisation of salinity time-series data and statistical models indicate positive associations between tropical cyclones and soil and water salinity. The impact of some cyclones on soil salinity and river water salinity is visible in monthly time-series data (see Fig. 2 and Fig. S1). For example, short-term deflections in monthly salinity time-series data can be seen in soil salinity at Krishnanagar (Fig. 2e) following cyclones Aila (2009) and Fani (2019). Similarly, deflections can be seen in surface water salinity data in River Rupsha (Fig. 2f) during cyclone Amphan (2020). The impacts of river water salinity could be better observed in daily monitoring data that do not exist in coastal Bangladesh. We noticed from the time-series records that river water and soil salinities return to background levels within a month or so. The response could be, however, different in the soil and surface water salinity in areas that are located within coastal dykes or embankments (land area inside dykes is as the polder). Recent studies^30^ report contamination of freshwater ponds due to storm surge inundation and breaching of protective earthen polders.

How do tropical cyclones and local weather influence soil and river water salinity? We examined the timing of tropical cyclones and their impacts on soil and river water salinity. Close inspection of the timing of cyclones and their signature on soil and river water salinity data reveal short-term deflections in salinity values that are short-lived but sufficient to increase soil salinity in open floodplains and in case of polder breaches. This is consistent with the regional-scale analysis of river water salinity in coastal Bangladesh^10^. Statistical models clearly suggest a positive association between cyclones and soil salinity (Tables 1 and 2). Observations suggest that cyclones making landfalls during the late monsoon season (September to November) when sea-surface salinity is at its lowest level (~ 25,000 µS/cm), have lower impacts on soil and river water salinity than those making landfalls during the pre-monsoon (March to May) season when sea salinity is at its highest levels (~ 40,000 µS/cm). For example, Cyclone Aila (May 2009) is reported to have much greater impacts on surface water such as ponds^30^ and soil salinity compared to super-cyclone Sidr that made landfall in November 2007 due to polder breaches^31^. The backwater effects of the sea and tropical cyclones clearly highlight the complex interplay between marine and terrestrial salinity dynamics in coastal Bangladesh.

Our statistical modelling results reveal significant associations between seasonal sea surface salinity and sea levels on soil and river water salinity. Cross correlation between monthly sea levels and sea salinity shows a strongly negative (Pearson correlation, *r*=‒0.803, p value < 0.001) association. A positive association with seasonal sea surface salinity emphasises the argument above on the timing of tropical cyclones landfalls and their impacts on salinity. A negative association with seasonal sea-level anomaly suggests that seasonally rising sea levels (i.e., positive anomaly) during the monsoon season coincide with lower soil and river water salinity. Due to the highly seasonal nature of salinity data and the large variations in salinity levels, the gradual rise in sea-level anomaly has a minimal impact on seasonal salinity patterns. In fact, rising sea levels do not necessarily mean an increased sea-surface salinity^32,33^. In monsoon-dominated deltaic systems, freshwater discharge from runoff and direct rainfall dilutes salinity levels. For instance, in the Ganges-Brahmaputra-Meghna (GBM) delta, the monsoon season brings substantial freshwater input, which flushes salt in seawater and reduces sea-surface salinity^34^.

Our statistical modelling shows that soil and river water salinity are strongly and inversely related to upstream river discharge (in the Gorai River, an offshoot of the Ganges), while surface water levels show a positive interaction effect. These may suggest that a decrease in freshwater discharge in the upstream rivers can increase brackish-saline water through backflow effects within the tidal rivers in the coastal region of Bangladesh. The model reveals an interesting association between groundwater levels and salinity. A seasonal rise in groundwater levels (i.e., shallowing of the water table) in shallow, brackish- to saline-water aquifers can influence soil salinity through capillary rise^35^. In contrast, an inverse association between deepening groundwater table and river water salinity suggests that water salinity is at its higher levels when base-flow to rivers is small or even absent due to reversal in hydraulic gradients. These associations mainly explain the seasonal variability in soil and river water salinity. Our models, however, cannot explain the long-term dynamics in the soil or river water salinity in relation to river discharge or groundwater-level variations.

Overall, our data-driven analyses of long-term soil and river water salinity time-series data reveal complex seasonal patterns influenced by various factors. Through data visualisation, statistical analyses, and modelling, we examined long-term time-series data alongside a dozen related variables. This approach uncovered significant associations between salinity levels and local weather, climate, hydrology, and anthropogenic factors. Our observations show that while cyclones can cause rapid changes in salinity levels, the impact is usually short-lived, and its intensity is largely influenced by seasonal factors. Impacts of storm surge inundation from tropical cyclones are more pronounced in soil salinity than in river water salinity. This is because storm surge inundation causes occasional breaches of earthen polders^14^ creating prolonged water-logging conditions. As a result, higher soil salinity can be sustained for a long period of time (weeks to months) when polders are breached (i.e., earthen dykes are washed away) during tropical cyclones contaminating land and water inside them^15^. The land surface elevation within polders is often lower than the adjacent floodplains^36^. A significant consequence of this elevation loss was the failure of polders during Cyclone Aila in 2009, causing tidal inundation of large areas for up to two years^37^. Land subsidence rates are much higher in southwestern coastal Bangladesh, contributing to relative sea-level rise within the polder areas, where elevation has been declining due to the lack of active sedimentation^38,39^. However, our model did not include land subsidence as a factor to account for its influence on water and soil salinity due to lack of time-series of subsidence data.

In contrast, cyclone-induced rainfall and runoff negate the effects of saline-water intrusion within a very short period of time (i.e. within days to weeks) as observed in Pearl River Delta in China^40^. Our analysis shows that early monsoon cyclones (April-May) cause more severe soil and water salinity increases than late to post-monsoon cyclones (October-December), due to peak sea salinity levels during this period. For example, cyclones Aila (May 2009) and Amphan (May 2020) had long-lasting impacts on southwestern coastal Bangladesh and notably disrupted the expected seasonal salinity in surface water and soil in the southwestern coast^30^. Tropical cyclones are becoming very common in May (e.g., Roanu in 2016, Fani in 2019, Amphan in 2020, Yaas in 2022, Remal in 2024) as we find in the International Best Track Archive for Climate Stewardship (IBTrACS) database^41^. The occurrence of cyclones in the early monsoon season along with slowly rising sea-levels may have contributed to an increasing trend in soil salinity observed in the last decade. Further statistical analysis of historical cyclones and sea-level data along the entire coast is needed to confirm this relationship.

Our analysis found no significant overall trends in soil or river water salinity over the past 18 years (Jan 2004 to Jun 2022), as opposing trends (see Fig. 2f; declining trend between 2004 and 2014 and rising trend between 2015 and 2022) in different periods offset each other. Steadily rising trends are observed primarily in the dry-season soil salinity since 2012-14, consistent with Salehin et al. ^9^ and both rising summer temperatures (Figure S13) and lower rainfall (Figure S14) during the early monsoon season throughout the southwestern coastal Bangladesh. Substantial increases in peak monsoon rainfall, mainly in July, are aligned to the global amplification of precipitation extremes under global warming^42^ and help to buffer seasonal soil and river water salinity. Furthermore, rising trends in sea-level anomaly are inversely correlated to sea-surface salinity indicating the effect of an increased seasonal rainfalls from July to September diluting sea-water salinity near the coastline (Fig. 3c, d and e). These meteorological and climatic changes shape the seasonal dynamics of soil and river water salinity in southwestern coastal Bangladesh.

Our findings from the analysis of soil and surface water salinity in Bangladesh provide valuable insights into water and soil salinisation in other Asian mega-deltas and coastal regions globally, particularly where long-term monitoring of salinity is limited or non-existent. By leveraging rare salinity time-series data, our data-driven visualisations and statistical analyses highlight critical dynamics essential for soil and water management and inform climate adaptation strategies in Bangladesh. Our findings reveal significant spatial and seasonal variability in soil and surface water salinity, highlighting the need for targeted, site-specific adaptation strategies in coastal Bangladesh. For example, overall soil and river water salinity in southwest coastal areas is significantly higher and highly seasonal compared to south-central coastal region. Therefore, we argue that a 'one-size-fits-all’ approach is unsuitable given the diversity of salinity hazards across regions. For example, in areas with high soil salinity in southwestern areas, salt-tolerant varieties of crops, mulching^43^, and organic soil amendments^44^ can help reduce the negative impacts. Where surface water salinity shows greater seasonal variability with low levels during the wet season, especially in waterlogged areas in southwestern areas, tidal river management could bring more benefits to agriculture and drainage^45,46^, whereas, pond/channel water storage, and rainwater harvesting^47^ offer viable solutions for drinking water storage in the dry season. Effective adaptation requires integrating local salinity patterns with community knowledge and climate projections to support resilient water and soil management. It is also crucial to evaluate the existing salinity adaptation measures such as the Managed Aquifer Recharge^48,49^, rainwater harvesting^50,51^, saline-resistant crops^52,53^, and improved coastal embankment (i.e., polder) management^53,54^ to determine how effective these strategies are in mitigating both soil and water salinity, especially since our modelling reveals that no single factor (e.g., sea level rise or tropical cyclones) acts as the sole driver.

### Concluding remarks

Our study draws on nearly two decades of rare time-series data to characterise the dynamics of soil and river water salinity in the southwestern coastal region of Bangladesh. By addressing the three guiding questions, we highlight the mechanisms shaping seasonal and long-term patterns and their implications for salinity adaptation strategy and national policy.

 1. What drives the seasonal variation in soil and river water salinity?

Our data-driven analyses confirm that soil and river water salinity levels are strongly seasonal, rising gradually with increasing temperature and evapotranspiration during the dry months and falling sharply with the onset of monsoon rainfall and river discharge. Seasonal dilution from rainfall and freshwater inflows explains the rapid reduction in salinity each year, underscoring the dominant role of local weather and hydrological cycles in shaping soil and river-water salinity regimes.

 2. How do tropical cyclones and local weather influence soil and river water salinity?

Tropical cyclones contribute to short-term spikes in salinity, particularly when landfall occurs during the pre- to early-monsoon period, when sea-surface salinity is at its annual peak. The impacts are more pronounced for soil salinity – especially where polders are breached and waterlogging persists – than for river water, where elevated salinity often subsides rapidly. While cyclone-driven local rainfall and river discharge can counteract saline intrusion, the overall impacts of cyclones remains significant for local communities and ecosystems.

3. What are the impacts of rising sea levels, sea salinity, terrestrial hydrology, and climate variability?

Statistical models reveal positive associations between soil and river water salinity and sea-surface salinity, while seasonal sea-level rise during the monsoon is inversely related to salinity due to the overwhelming freshwater fluxes. Changes in river discharge and groundwater levels also interact with salinity dynamics, reflecting the delicate balance between saline water intrusion and terrestrial hydrological inputs. Although no consistent long-term trend is observed across the full 18-year record, rising dry-season soil salinity since the mid-2010s suggests that climate variability, reduced early-monsoon rainfall, land subsidence contributing to relative sea-level rise, and the timing of cyclones may be amplifying the salinity risks in recent years.

Taken together, our findings demonstrate that soil and river water salinity in coastal Bangladesh is controlled by a complex interplay of local seasonal weather, climate, hydrology, anthropogenic, and tropical cyclonic events. They highlight the need for assessing the regional-scale water and soil salinity risk as well as routine high-frequency monitoring and integrated modelling to anticipate water and soil salinity risks under changing climatic and land-use conditions. Salinity adaptation strategies – whether through salt-tolerant crops, improved polder embankment, and tidal water management, or rainwater harvesting – must be designed with a nuanced understanding of local salinity regimes in the coastal areas. In line with the long-term vision of the Bangladesh Delta Plan 2100, our results underscore the importance of integrating empirical monitoring with predictive modelling of coastal salinity issue so that policymakers and practitioners can better support climate-resilient water and agricultural management in Bangladesh and other vulnerable deltaic environments.

## Methods

### Soil and river water salinity datasets

We use long-term (January 2004 to December 2022) monthly time-series salinity data from 11 soil salinity and 13 river water salinity stations in southwestern coastal Bangladesh. These monitoring stations are located within three coastal districts of Bangladesh namely Bagerhat, Khulna and Satkhira (Fig. 1b).

The monthly time-series data were collated from the local office of the Soil Resource Development Institute (SRDI) in Khulna, Bangladesh. SRDI is a government organisation operating under the Ministry of Agriculture. SRDI collects information on soil and water properties for sustainable agricultural production through improved management of soil as well as preservation of environment. To analyse seasonal variation in soil and water salinity, SRDI collects monthly soil samples from 11 stations and river water samples from 13 stations in southwestern Bangladesh which is one of the most salinity-affected areas in the country. Seven of the 11 stations of soil salinity have monthly data from January 2004 to June 2022, and the remaining 4 stations have soil salinity data from January 2018 to June 2022, whereas all the 13 stations of surface water salinity have monthly data from January 2004 to June 2022.These rare time-series data of soil and water salinity have not previously been analysed or published.

Monthly soil salinity at the 11 stations derives from soil samples from the respective locations. Samples are then tested for the Electrical Conductivity (EC) of a saturated soil Extract (ECe), a reliable method for measuring soil salinity^55^. In this sampling, soils are collected from 0 to 15 cm depth of the soil profile. The collected soil samples are first air-dried and cleared from unwanted materials such as plant remains. Once the sample is properly dried, wooden hammer is used to break down the larger aggregates into smaller particles. The powdered soil sample is then filtered through a 2 mm sieve. This filtered soil is added to distilled water maintaining a ratio of 1:5 to fill the available pores and then is mixed to the consistency of a paste that glistens with water and if jarred flows slightly. After allowing sufficient time, ideally overnight for the soil to equilibrate and dissolve the salts thoroughly, the resulting water is extracted by suction filtration and Electrical Conductivity (EC) is determined by a portable EC meter. However, saturated extract (soil: water = 1:1) produces the best result for soil EC but preparation of saturated extract is cumbersome. Therefore, a ratio of 1:5 (soil: water) is used and the result is converted into saturated extract (ECe).

In case of surface water salinity, river water was collected in plastic bottles after rinsing it with sample water for about two to three times. The samples were labelled with sample identification (ID) and the information about the sampling points are recorded. After the collection of water samples, all bottles were sealed immediately to avoid exposure to air. After labelling, water samples were transported to the laboratory of SRDI following sampling protocols from sampling to transportation. All the samples were filtered through filter paper (Whatman No.1) to remove unwanted solid and suspended materials before analysis. The samples were then measured by an EC meter.

### Monthly rainfall and temperature time-series data

We collated daily temperature and rainfall time-series data from the Bangladesh Meteorological Department (BMD) at three stations (Khulna, Satkhira and Mongla) located in the southwestern Bangladesh. BMD is a government meteorological agency that has been collecting data since 1950s. We collated the available daily time-series data (January 2004 to December 2021) at three stations and converted it to monthly data for statistical analysis and modelling in this study. Monthly rainfall and temperature data for 2022 at the three BMD stations’ locations were downloaded from the TerraClimate product^56^ available on an online data portal called Climate Engine (https://www.climateengine.org)^57^. We also collated monthly evapotranspiration and Normalised Difference Vegetation Index (NDVI) data at the selected BMD locations from the NASA’s Famine Early Warning Systems Network (FEWS NET) Land Data Assimilation System (FLDAS) via the Climate Engine data portal.

### Hydrological and hydrogeological data

We collated monthly time-series data for surface runoff and average soil moisture at the selected three BMD locations (Khulna, Satkhira and Mongla) from the NASA’s Famine Early Warning Systems Network (FEWS NET) Land Data Assimilation System (FLDAS) via the Climate Engine data portal. River discharge, surface water level and groundwater level data were collated from the Bangladesh Water Development Board (BWDB). Average river discharge data derived from two BWDB stations: SW90 (Ganges-Padma River at Hardinge Bridge) and SW99 (Gorai-Madhumati River at Gorai Railway Bridge). We also used SW241 data as a covariate for helping with data extrapolation and imputation. Average surface water-level data derived from 11 BWDB stations: SW1, SW24, SW28, SW105, SW107.2, SW241, SW243, SW244, SW254.5, SW258 and SW259. We used time-series data (2008–2022) from two virtual stations (IDs: 3190 and 3191) within the Database for Hydrological Time Series of Inland Waters (DAHITI) data portal (available at https://dahiti.dgfi.tum.de/en/map/) as an additional variable for surface water-level data imputation. Finally, average groundwater-level data derived from four BWDB boreholes: KH003, KH005, KH008 and KH021.

### Sea level anomaly and sea surface salinity data

We used monthly sea level anomaly and sea surface salinity data from the Copernicus Marine data services through the data visualisation tool called My Ocean Pro portal (https://marine.copernicus.eu/access-data/myocean-viewer). Monthly gridded (0.25° × 0.25° spatial resolution) mean of Sea Level Anomalies (SLA) derived from altimeter satellite measurements computed with respect to a 20-year (1993–2012) mean. The sea-level anomalies (Product: SEALEVEL_GLO_PHY_L4_MY_008_047) were estimated by an Optimal Interpolation method that merged the Level 3 (L3) along-track measurement from different altimeter satellite missions^67^. The sea surface salinity gridded (0.083° × 0.083°) monthly mean data derived from numerical models under the Global Ocean Physics Reanalysis product (GLOBAL_MULTIYEAR_PHY_001_030^58^;. Both SLA and sea salinity data were downloaded for the upper portion of the Bay of Bengal (north of 19° latitude and within 86° and 95° E longitudes) and aggregated monthly time-series data were generated using all the available grid points. Gridded data were processed in R programming language^59^ using various packages.

### Data processing and exploratory analysis

Soil and river water salinity time-series data were collated in MS Word format from SRDI, Khulna. Daily rainfall and temperature data were collated in MS Excel format from BMD. All time-series data are then converted to a standard MS Excel comma-separated values (CSV) file format, processed and analysed in R programming language. Both R GUI (version 4.4.2; https://www.r-project.org/) and RStudio (2024.12.0 + 467; https://posit.co/download/rstudio-desktop/) software were used for data processing and statistical analyses. All time-series data are converted to mean monthly values for consistency (total number of months = 222). The proportion of missing values in the datasets is as follows: average soil and river water salinity data (4%); no missing values for average rainfall, evapotranspiration, temperature, soil moisture, runoff, number of cyclone, sea level anomaly, and sea surface salinity time-series data; river discharge (35%), surface water levels (20%), groundwater levels (19%) and NDVI (38%). Geospatial data were processed and visualised using ESRI’s ArcGIS software (ArcGIS Desktop version 10.8; https://www.esri.com/en-us/store/overview).

For statistical modelling, all missing data in the response variables and covariate time-series records were imputed or infilled using Random Forest machine learning algorithm from the missForest package in R language (Stekhoven and Bühlmann, 2012). Random Forest algorithm, for imputation purposes, does not require a validation dataset in this case, as it uses a technique called out-of-bag (OOB) to evaluate the quality of the imputation model in this case. OOB evaluation treats the training dataset as if it were on the test dataset of a cross-validation.

Performance of data imputation by missForest package is evaluated by the normalised root mean squared error (NRMSE), which is defined in Eq. 1. The overall imputation error is 11.5% (NRMSE) which is low and reasonable given the high range of missing values in some covariate (river discharge: 35%) datasets.

1 $$\:\sqrt{\frac{mean\:{\left({X}_{true}-{X}_{imp}\right)}^{2}}{var\left({X}_{true}\right)}}$$

Where $\:{X}_{true}$ is the complete data matrix, $\:{X}_{imp}$ is the imputed data matrix and mean/var being used as short notation in Eq. 1 for the empirical mean and variance computed over the continuous missing values only.

### Trend analysis using parametric and non-parametric methods

Descriptive statistics from the 24 time-series (Jan 2004 to Dec 2022) salinity data are calculated in R platform. We apply both parametric and non-parametric trend analyses and decomposed the monthly series into seasonal, trend and residual components using the Seasonal and Trend Decomposition using Loess (STL) method (Cleveland et al., 1990). STL is well-suited for environmental data as it handles non-linear trends and varying seasonal patterns. This decomposition helps distinguish long-term changes from recurring seasonal effects and short-term fluctuations. To assess the relative importance of each component, we calculated the proportion of variance explained by the trend, seasonal, and irregular parts at each station. These proportions reveal which sources of variability dominate across sites. In addition, for the trend analysis, we apply linear trend, Mann-Kendall (MK) trend test, seasonal MK trend, Sen’s slope and seasonal Sen’s slope^27,60^.

### Cross-correlation and wavelet analysis

As a part of our exploratory analysis, we examined the correlation and cross-correlation between soil and river water salinity along with 12 covariates using the Pearson correlation and Cross Correlation Function (CCF) in R GUI environment (R version 4.4.2). We approximately grouped the covariate datasets into hydrological factors (i.e., evapotranspiration, river discharge, surface water levels, soil moisture, groundwater levels), meteorological (i.e., rainfall, temperature, occurrence of cyclones), climatological (i.e., sea level anomaly, sea surface salinity), and anthropological (i.e., normalised difference vegetation index or NDVI as an indicator of land-use changes).

We apply wavelet analysis using the WaveletComp^61^, an R package for continuous wavelet-based analysis of soil and river water salinity and meteorological time series records. Wavelet analysis is a powerful tool that decomposes a time series into time-frequency space, allowing the examination of how periodic components of the data evolve over time^62^. When applied to a single time series, wavelet analysis reveals the presence and strength of periodicities (cyclical patterns) at different scales (frequencies) and how these periodicities change over time which makes it particularly useful for identifying localised events or changes in cyclic behaviour^26^. For coherence analysis between two time series, the cross-wavelet transforms, and wavelet coherence techniques are used^62^.

### Statistical modelling

In this study, we develop statistical models to explain the variability observed in the time-series data of soil and river water salinity in coastal Bangladesh using twelve covariates representing hydrological (i.e., meteorological, climatological and anthropogenic processes. We use Multiple Linear Regression (MLR) to model the statistical variability in the response or dependent variables such as the soil salinity and river water salinity. The MLR model take the following form in Eq. (1).

2 $$\:\log \:\left( y \right) = \beta \:_{0} + \beta \:_{1} x_{1} + \beta \:_{2} x_{2} + \ldots \: + \:\beta \:_{n} x_{n} + \:\varepsilon \:$$

The response variable $\:\text{l}\text{o}\text{g}\left(y\right)\:$(soil or river water salinity) is log-transformed (natural logarithm) due to the skewness in the original data distribution. In Eq. (2), $\:{\beta\:}_{0},\:{\beta\:}_{1},\:{\beta\:}_{2},\:{\dots\:\beta\:}_{n\:}$are model coefficients, $\:{x}_{1\:},\:{x}_{2\:},\:\dots\:{x}_{n\:}$are predictor or covariate datasets ($\:n=14)$, and $\varepsilon$ represents the error term in the model. This means that a 1-unit increase in$\:\:{x}_{\:}$will lead to a proportional change in $\:y$, so $\:\%\varDelta\:y=\left({e}^{{\beta\:}_{1}}-1\right)\times\:100.\:$We use dynamic linear regression in ‘dynlm’ R package for modelling soil and river water salinity^63^.

Exploratory analysis shows that the time-series data of response variables and covariates have high seasonality, and some are highly correlated to each other raising the concern of multicollinearity. This occurs when two or more predictors in a regression model are highly correlated. Multicollinearity can cause the estimates of the regression coefficients to become unstable and highly sensitive to small changes in the model or data that can result in large standard errors and less reliable coefficient estimates^64^. We check correlation matrix and Variance Inflation Factor (VIF) in the fitted model to consider dropping some variables that can cause multicollinearity effects. VIF values greater than 10 are commonly used as a rule-of-thumb to indicate potential multicollinearity among predictors in a regression model. While assessing multicollinearity using VIF, we found acceptable levels for rainfall (VIF = 8.6) and surface runoff (VIF = 5.7); however, a strong Pearson correlation (*r* = 0.88, *p* value = < 0.001) exists between these two variables. Given their hydrological interdependence and the risk of inflated standard errors and unstable coefficient estimates, we decided to exclude surface runoff from the final statistical models. This step improves the robustness and interpretability of the regression results, particularly for isolating the effect of rainfall on the outcome variable.

We also consider adding interaction terms to the model to explore joint effects on the response variable. The fitted model is diagnosed using standard plots in R GUI (R version 4.4.2), and model summary statistics are derived from the final model for interpretation. Two separate MLR models are created using the same set of covariates to explain the variability observed in soil and river water salinity datasets. MLRs are created with individual sets of covariates representing meteorological, climatological, hydrological and hydrogeological and land-use/land-cover and anthropogenic factors).

In addition to MLR models, we apply Generalised Linear Models (GLMs)^65^ to explain the variability in soil and river water salinity in southwest coastal Bangladesh. The GLM takes the following form in Eq. (3) with a link function $\:\mathbf{g}\left(\mu\:\right)=\eta\:$ and $\:\mu\:=\mathbb{E}\left(\varvec{y}\right)$ is the is the expected value of the response variable.

3 $$\:g({\mathbb{E}}(y)) = \beta \:_{0} + \beta \:_{1} x_{1} + \beta \:_{2} x_{2} + \ldots \: + \:\beta \:_{n} x_{n}$$

In this case, soil or river water salinity being the response variable the final form of the GLM can be expressed as Eq. (4) with Gamma-distributed outcome and a log link:

4 $$\:\log \left( {\mu \:} \right) = \beta \:_{0} + \beta \:_{1} AvgRain + \beta \:_{2} AvgTemp + \ldots \: + \:\beta \:_{n} x_{n}$$

In these formulae, *µ* is the mean of the response variable, *g*(⋅) is the link function and *β* is the vector of regression coefficients. We use glm() function in R programming language to create models to explain soil and river water salinity.

#### Limitations and sources of uncertainty

This study on soil and surface water salinity in southwestern Bangladesh is subject to several limitations. First, the analysis is based on a relatively small number of long-term monitoring stations: 11 for soil salinity and 13 for river water salinity, covering the period from January 2004 to June 2022. All stations are located in a region where salinity levels are known to be high, limiting the geographic representativeness of the findings.

Although the time series spans 18.5 years, there are data gaps in 9 out of the 222 months, which may have affected the continuity of statistical analysis. Furthermore, the salinity measurements – conducted by the Soil Resource Development Institute (SRDI), Bangladesh – may be subject to instrument or procedural uncertainties inherent in field-based data collection over extended periods.

In terms of modelling, we used twelve covariates derived from multiple secondary sources. Notably, several monthly variables including evapotranspiration, surface runoff, soil moisture, and NDVI were sourced from the FLDAS land surface model, which is known to contain inherent uncertainties typical of such global-scale datasets. Due to limited time-series data availability, we could not assess the impact of land subsidence on salinity, which we think is quite significant in southwestern coastal Bangladesh. .

Finally, to address missing values in both the response variables and covariates, we employed random forest imputation methods within the R programming environment (missForest R package). While this approach allowed for a complete dataset suitable for modelling, it introduced additional uncertainty, particularly as imputation inherently involves assumptions about data structure and distribution. However, we have confidence in the imputation process as the error is reported to be low (normalised root mean squared error: 11.5%).

## Supplementary Information

Below is the link to the electronic supplementary material.

Supplementary Material 1

## Data Availability

Monthly time-series data on soil and surface-water salinity (2004–2022), collated from the Soil Resource Development Institute (SRDI), Bangladesh, together with the variables used in the statistical models, are available from the corresponding author upon reasonable request. We do not have permission from SRDI Bangladesh to make these monitoring data publicly available. All statistical analyses and modelling were conducted in the R programming environment (R GUI version 4.4.2 and RStudio version 2024.12.0+467), and the R scripts are available from the corresponding author for researchers interested in reproducing the results of this study.

## References

[1] Mukhopadhyay, R., Sarkar, B., Jat, H. S., Sharma, P. C. & Bolan, N. S. Soil salinity under climate change: challenges for sustainable agriculture and food security. *J. Environ. Manage.* **280**, 111736 (2021). 33298389 10.1016/j.jenvman.2020.111736

[2] Negacz, K., Malek, Ž., de Vos, A. & Vellinga, P. Saline soils worldwide: identifying the most promising areas for saline agriculture. *J. Arid Environ.* **203**, 104775 (2022).

[3] Kumar, P. & Sharma, P. K. Soil salinity and food security in India. *Front. Sustainable Food Syst.* **4**, 533781 (2020).

[4] Qureshi, A. S., McCornick, P. G., Sarwar, A. & Sharma, B. R. Challenges and prospects of sustainable groundwater management in the indus Basin, Pakistan. *Water Resour. Manage*. **24**, 1551–1569 (2010).

[5] Morton, L. W., Nguyen, N. K. & Demyan, M. S. Salinity and acid sulfate soils of the Vietnam Mekong delta: agricultural management and adaptation. *J. Soil Water Conserv.* **78**, 85A–92A (2023).

[6] Yu, J. et al. The Spatial distribution characteristics of soil salinity in coastal zone of the yellow river delta. *Environ. Earth Sci.* **72**, 589–599 (2014).

[7] Shawkhatuzamman, M. et al. Soil salinity management practices in coastal area of bangladesh: a review. *Res. Agric. Livest. Fisheries*. **10**, 1–7 (2023).

[8] Sahana, M. et al. Assessing the degree of soil salinity in the Indian Sundarban biosphere reserve using measured soil electrical conductivity and remote sensing data–derived salinity indices. *Arab. J. Geosci.* **13**, 1–15 (2020).

[9] Salehin, M. et al. Mechanisms and drivers of soil salinity in coastal Bangladesh *in Ecosystem Services for Well-Being in Deltas* (eds. Nicholls, R. et al.) 333–347 (Palgrave Macmillan, 2018).

[10] Haq, M.I., Shamsudduha, M., Zahid, A., Ahmed, K.M., Kamal, A.M. & Taylor, R.G. What drives changes in surface water salinity in coastal Bangladesh? *Front. Water*. **6**, 1220540 (2024).

[11] Feist, S. E., Hoque, M. A. & Ahmed, K. M. Coastal salinity and water management practices in the Bengal delta: A critical analysis to inform salinisation risk management strategies in Asian deltas. *Earth Syst. Environ.* **7**, 171–187 (2023).

[12] Chowdhury, M. A., Khairun, Y., Salequzzaman, M. & Rahman, M. M. Effect of combined shrimp and rice farming on water and soil quality in Bangladesh. *Aquacult. Int.* **19**, 1193–1206 (2011).

[13] Clarke, D., Williams, S., Jahiruddin, M., Parks, K. & Salehin, M. Projections of on-farm salinity in coastal Bangladesh. *Environ. Science: Processes Impacts*. **17**, 1127–1136 (2015). 10.1039/c4em00682h 25790459

[14] Islam, M. F., Bhattacharya, B. & Popescu, I. Flood risk assessment due to cyclone-induced dike breaching in coastal areas of Bangladesh. *Nat. Hazards Earth Syst. Sci.* **19**, 353–368 (2019).

[15] Bhuyan, M. I., Mia, S., Supit, I. & Ludwig, F. Spatio-temporal variability in soil and water salinity in the south-central Coast of Bangladesh. *Catena* **222**, 106786 (2023). 10.1016/j.heliyon.2023.e19180 PMC10469569 37664704

[16] Kawser, U., Nath, B. & Hoque, A. Observing the influences of Climatic and environmental variability over soil salinity changes in the Noakhali coastal regions of Bangladesh using Geospatial and statistical techniques. *Environ. Challenges*. **6**, 100429 (2022).

[17] Rahman, M. O. & Rahman, A. K. M. A. Adaptation strategy with climate induced salinity disaster in the coastal area of Bangladesh. *Am. J. Clim. Change*. **11**, 284–306 (2022).

[18] Jahan, K., Zahid, A., Bhuiyan, M. A. E. & Ali, I. A resilient and nature-based drinking water supply source for saline and arsenic prone coastal aquifers of the Bengal delta. *Sustainability* **14**, 6703 (2022).

[19] Sahbeni, G. & Székely, B. Salinity Levels Discrimination Using ERS-1/2 and Sentinel-1 SAR Time Series Data in Hortobágyi National Park, Hungary *in IEEE Mediterranean and Middle-East Geoscience and Remote Sensing Symposium (M2GARSS)* 194–197 (IEEE, 2022).

[20] Taghadosi, M. M. & Hasanlou, M. Trend analysis of soil salinity in different land cover types using Landsat time series data (case study Bakhtegan salt Lake). *Int. Archives Photogrammetry Remote Sens. Spat. Inform. Sci.* **42**, 251–257 (2017).

[21] Bandak, S., Movahedi-Naeini, S. A., Mehri, S. & Lotfata, A. A longitudinal analysis of soil salinity changes using remotely sensed imageries. *Sci. Rep.* **14**, 10383 (2024). 38710771 10.1038/s41598-024-60033-6 PMC11074301

[22] Bannari, A. & Al-Ali, Z. M. Assessing climate change impact on soil salinity dynamics between 1987–2017 in arid landscape using Landsat TM, ETM + and OLI data. *Remote Sens.* **12**, 2794 (2020).

[23] Sarkar, S. K. et al. Coupling of machine learning and remote sensing for soil salinity mapping in coastal area of Bangladesh. *Sci. Rep.* **13**, 17056 (2023). 37816754 10.1038/s41598-023-44132-4 PMC10564761

[24] Eltarabily, M. G. et al. Time-Lapse electromagnetic conductivity imaging for soil salinity monitoring in Salt-Affected agricultural regions. *Land* **13**, 225 (2024).

[25] Cleveland, R. B., Cleveland, W. S., McRae, J. E. & Terpenning, I. STL: A seasonal-trend decomposition. *J. Off Stat.* **6**, 3–73 (1990).

[26] Percival, D. B. & Walden, A. T. *Wavelet Methods for time Series Analysis* Vol. 4 (Cambridge University Press, 2000).

[27] Hirsch, R. M., Slack, J. R. & Smith, R. A. Techniques of trend analysis for monthly water quality data. *Water Resour. Res.* **18**, 107–121 (1982).

[28] Hossain, M. K. et al. Geospatial analysis of soil salinity dynamics: Exploring topographic and vegetation influences in coastal Bangladesh. *Geosyst. Geoenviron.* **4**, 100418 (2025).

[29] Shamsudduha, M., Taylor, R. G. & Chandler, R. E. A generalized regression model of arsenic variations in the shallow groundwater of Bangladesh. *Water Resour. Res.* **51**, 685–703 (2015). 27524841 10.1002/2013WR014572 PMC4964952

[30] Tsai, C., Hoque, M. A., Vineis, P., Ahmed, K. M. & Butler, A. P. Salinisation of drinking water ponds and groundwater in coastal Bangladesh linked to tropical cyclones. *Sci. Rep.* **14**, 5211 (2024). 38433257 10.1038/s41598-024-54446-6 PMC10909877

[31] Mainuddin, M. & Kirby, J. M. Impact of flood inundation and water management on water and salt balance of the polders and Islands in the Ganges delta. *Ocean. Coastal. Manage.* **210**, 105740 (2021). 10.1038/s41598-021-86206-1 PMC8007752 33782450

[32] Durack, P. J., Wijffels, S. E. & Gleckler, P. J. Long-term sea-level change revisited: the role of salinity. *Environ. Res. Lett.* **9**, 114017 (2014).

[33] Cheng, L. et al. Improved estimates of changes in upper ocean salinity and the hydrological cycle. *J. Clim.* **33**, 10357–10381 (2020).

[34] Bricheno, L. M., Wolf, J. & Sun, Y. Saline intrusion in the Ganges-Brahmaputra-Meghna megadelta. *Estuar. Coast. Shelf Sci.* **252**, 107246 (2021).

[35] Jorenush, M. & Sepaskhah, A. Modelling capillary rise and soil salinity for shallow saline water table under irrigated and non-irrigated conditions. *Agric. Water Manage.* **61**, 125–141 (2003).

[36] Zaman, S. & Mondal, M. S. Risk-based determination of polder height against storm surge hazard in the south-west coastal area of Bangladesh. *Progress Disaster Sci.* **8**, 100131(2020).

[37] Auerbach, L. W. et al. Flood risk of natural and embanked landscapes on the Ganges–Brahmaputra tidal delta plain. *Nat. Clim. Change*. **5**, 153–157 (2015).

[38] Steckler, M. S. et al. Contribution of campaign GNSS toward parsing subsidence rates by time and depth in coastal Bangladesh. *Front. Earth Sci.* **12**, 1354686 (2024).

[39] Steckler, M. S. et al. Synthesis of the distribution of subsidence of the lower Ganges-Brahmaputra Delta, Bangladesh. *Earth Sci. Rev.* **224**, 103887 (2022).

[40] Gao, Y. et al. Characteristics and influencing factors of storm Surge-Induced salinity augmentation in the Pearl river Estuary, South China. *Sustainability* **16**, 2254 (2024).

[41] Gahtan, U. Encrypted AI for environmental monitoring systems. *Int. J. Res. Radicals Multidisciplinary Fields* **3**, 24–32 (2024).

[42] Douville, H. et al. Water Cycle Changes *in Climate Change 2021: The Physical Science Basis* (eds. Masson-Delmotte, V. et al.), Intergovernmental Panel on Climate Change, Cambridge, United Kingdom and New York, NY, USA (2021).

[43] Ahmad, A., Blasco, B. & Martos, V. Combating salinity through natural plant extracts based biostimulants: a review. *Front. Plant Sci.* **13**, 862034 (2022). 35668803 10.3389/fpls.2022.862034 PMC9164010

[44] Shrivastava, P. & Kumar, R. Soil salinity: a serious environmental issue and plant growth promoting bacteria as one of the tools for its alleviation. *Saudi J. Biol. Sci.* **22**, 123–131 (2015). 25737642 10.1016/j.sjbs.2014.12.001 PMC4336437

[45] Al Masud, M. M., Värnik, R., Dogot, T. & Azadi, H. Sustainability index of tidal river management: A framework for measuring water sustainability in coastal areas. *Water* **17**, 648 (2025).

[46] Al Masud, M. M., Gain, A. K. & Azad, A. K. Tidal river management for sustainable agriculture in the Ganges-Brahmaputra delta: implication for land use policy. *Land. Use Policy*. **92**, 104443 (2020).

[47] Abdullah, M., Idrak, F., Kabir, P. & Bhuiyan, M. Suitability of rainwater harvesting in saline and arsenic affected areas of Bangladesh. *Heliyon* **10**, e34328 (2024). 39108884 10.1016/j.heliyon.2024.e34328 PMC11301181

[48] Naus, F. L., van Koppen, K., van der Zaag, P. & Sutherland, A. J. Why do people remain attached to unsafe drinking water options? Quantitative evidence from Southwestern Bangladesh. *Water* **12**, 342 (2020).

[49] Shammi, M., Rahman, M. M., Bondad, S. E. & Bodrud-Doza, M. Impacts of Salinity Intrusion in Community Health: A Review of Experiences on Drinking Water Sodium from Coastal Areas of Bangladesh. *Healthcare (MDPI)* **7**, 50 (2019). 10.3390/healthcare7010050 PMC6473225 30909429

[50] Ashrafuzzaman, M., Gomes, C. & Guerra, J. The changing climate is changing safe drinking Water, impacting health: A case in the Southwestern coastal region of Bangladesh (SWCRB). *Climate* **11**, 146 (2023).

[51] Islam, M. S., Deb, B. K., Alam, S. R. & Nishat, A. Technology for adaptation: a case study of developing a detailed inventory of drinking water supply technologies along the salinity-affected coastal region of Bangladesh. *AQUA-Water Infrastructure Ecosyst. Soc.* **72**, 673–689 (2023).

[52] Paul, B. & Rashid, H. Climatic hazards in coastal Bangladesh: non-structural and structural solutions. (Butterworth-Heinemann, 2016).

[53] Islam, M. F., Middelkoop, H., Schot, P. P., Dekker, S. C. & Griffioen, J. Enhancing effectiveness of tidal river management in Southwest Bangladesh polders by improving sedimentation and shortening inundation time. *J. Hydrol.* **590**, 125228 (2020).

[54] Rahman, S. M., Rahman, S. M. & Islam, M. B. Coastal Dynamics and Polder Management in the Context of Climate Change in the Southern Part of Bangladesh *in Handbook of Climate Change Management* (eds. Luetz, J.M. & Ayal, D.) 2889–2910 (Springer, 2021).

[55] Jones, C. The great salinity debate: part III. Soil organic matter: past lessons for future learning. *Stipa Newsletter*, 4–9 (2001).

[56] Abatzoglou, J. T., Dobrowski, S. Z., Parks, S. A. & Hegewisch, K. C. TerraClimate, a high-resolution global dataset of monthly climate and Climatic water balance from 1958–2015. *Sci. Data*. **5**, 1–12 (2018). 29313841 10.1038/sdata.2017.191 PMC5759372

[57] Huntington, J. L. et al. Climate engine: cloud computing and visualization of climate and remote sensing data for advanced natural resource monitoring and process Understanding. *Bull. Am. Meteorol. Soc.* **98**, 2397–2410 (2017).

[58] Drévillon, M. et al. Global Ocean Reanalysis Products GLOBAL_REANALYSIS_PHY_001_030. EU Copernicus Marine Service **1.6** (2022).

[59] R Core Team. *R: A Language and Environment for Statistical Computing. *R Foundation for Statistical Computing, Vienna, Austria (2022).

[60] Hipel, K. W. & McLeod, A. I. Time series modelling of water resources and environmental systems (Elsevier, 1994).

[61] Rösch, A. & Schmidbauer, H. *WaveletComp: Computational Wavelet Analysis. *R package version 1.1 (2018).

[62] Jevrejeva, S., Moore, J. & Grinsted, A. Oceanic and atmospheric transport of multiyear El nino–southern oscillation (ENSO) signatures to the polar regions. *Geophysical Res. Lett.* **31**, (2004).

[63] Zeileis, A. et al. colorspace: A toolbox for manipulating and assessing colors and palettes. *arXiv preprint arXiv:1903.06490* (2019).

[64] Kutner, J. S. et al. Outcomes and characteristics of patients discharged alive from hospice. *J. Am. Geriatr. Soc.* **52**, 1337–1342 (2004). 15271123 10.1111/j.1532-5415.2004.52365.x

[65] Chandler, R. E. & Wheater, H. S. Analysis of rainfall variability using generalized linear models: A case study from the West of Ireland. *Water Resour. Res.* **38**, 1192 (2002).

[66] Lassiter, A. Rising seas, changing salt lines, and drinking water salinization. *Current Opinion in Environmental Sustainability*, **50**, 208–214 (2021). 10.1016/j.cosust.2021.04.009

[67] Pujol M-I, et al. Refining the Resolution of DUACS Along-Track Level-3 Sea Level Altimetry Products. *Remote Sensing*, **15**(3), 793 (2023).

