# Supplementary material for: Soil and river water salinity dynamics in coastal Bangladesh
Source: Sci Rep. 2025 Dec 17;16:1044. doi: 10.1038/s41598-025-30639-5 (PMC12783709; doi:10.1038/s41598-025-30639-5)
Supplement: Supplementary file 1 — Supplementary Material 1 [file 41598_2025_30639_MOESM1_ESM.pdf]

SCIENTIFIC REPORTS

Soil and River Water Salinity Dynamics in Coastal Bangladesh

Ahmed Z. Rahman[1,2,*], Mohammad Shamsudduha[1], Md Izazul Haq[1,3,4], Md Hanif[5], Md Sanaul Islam[5], Syed S. Islam[5], Amarendranath Biswas[6], Richard G. Taylor[3]

[1]Department of Risk and Disaster Reduction, University College London, London, UK
[2]Ministry of Public Administration, Bangladesh Secretariat, Dhaka, Bangladesh
[3]Department of Geography, University College London, London, UK
[4]Department of Disaster Science and Climate Resilience, University of Dhaka, Dhaka, Bangladesh
[5]Soil, Water and Environment Discipline, Khulna University, Khulna, Bangladesh
[6]Salinity Management and Research Centre, Soil Resource Development Institute, Khulna, Bangladesh

*Corresponding author: ahmed.rahman.21@ucl.ac.uk

Supplementary Information

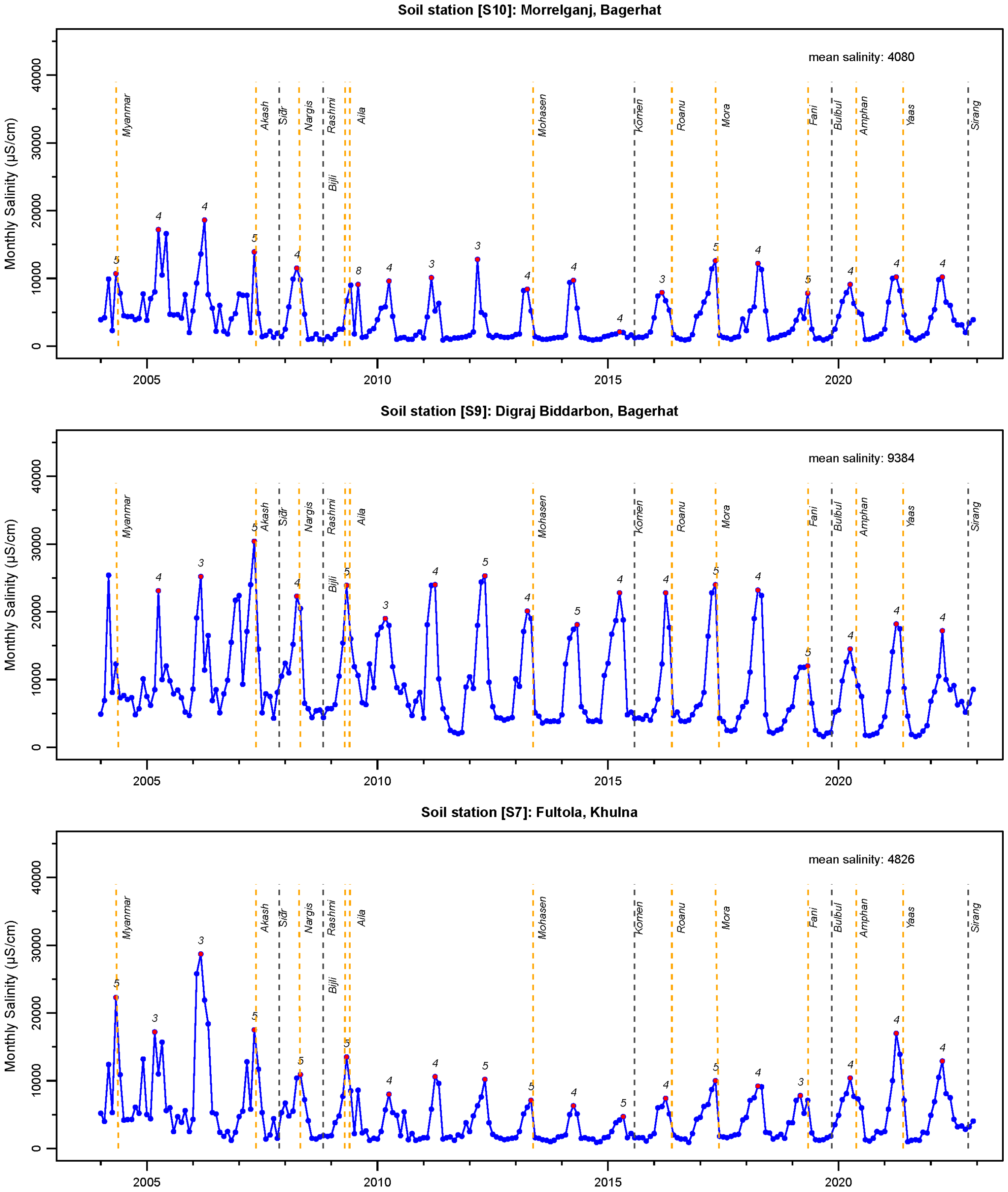

Figure S1. Time-series plots of monthly soil (*n*=7) and surface water or river water (*n*=11) salinity in southwestern coastal Bangladesh. Salinity is being monitored at 11 soil sampling sites and 13 river stations by the Soil Resource Development Institute (SRDI), Bangladesh. However, data from stations with continuous long-term (2004-2022) records are presented here. The month of peak seasonal salinity in each year is marked with red solid circles. Plots of monthly monitoring records of soil and river water salinity are combined with timing of tropical cyclones shown by vertical dash lines. The orange vertical dash lines indicate cyclones making landfall in April and May, and the grey lines indicate cyclones making landfall during and after the monsoon season. Mean salinity of the entire monitoring period is provided on the top-right corner of each time-series plot.

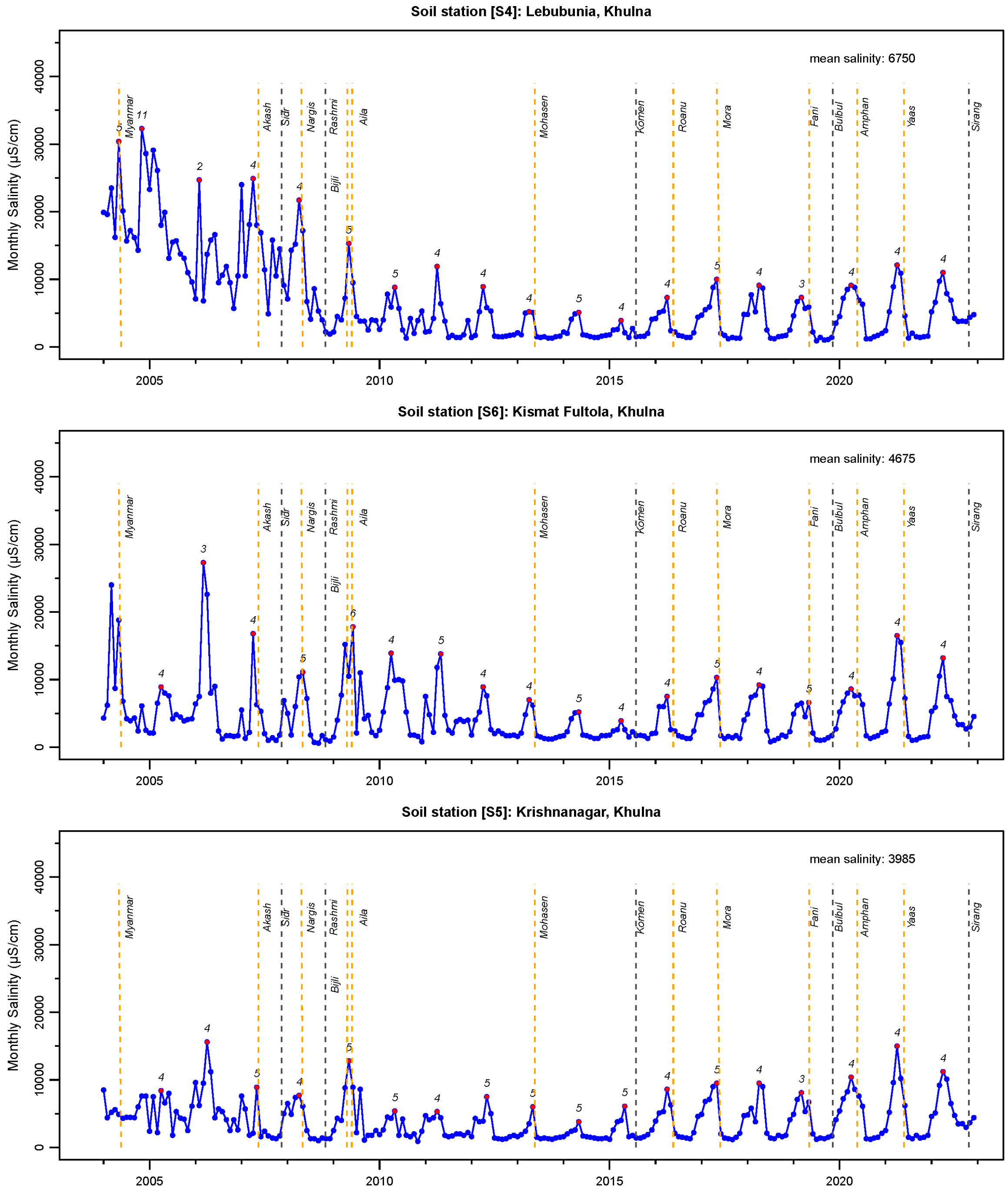

Figure S1. Continued.

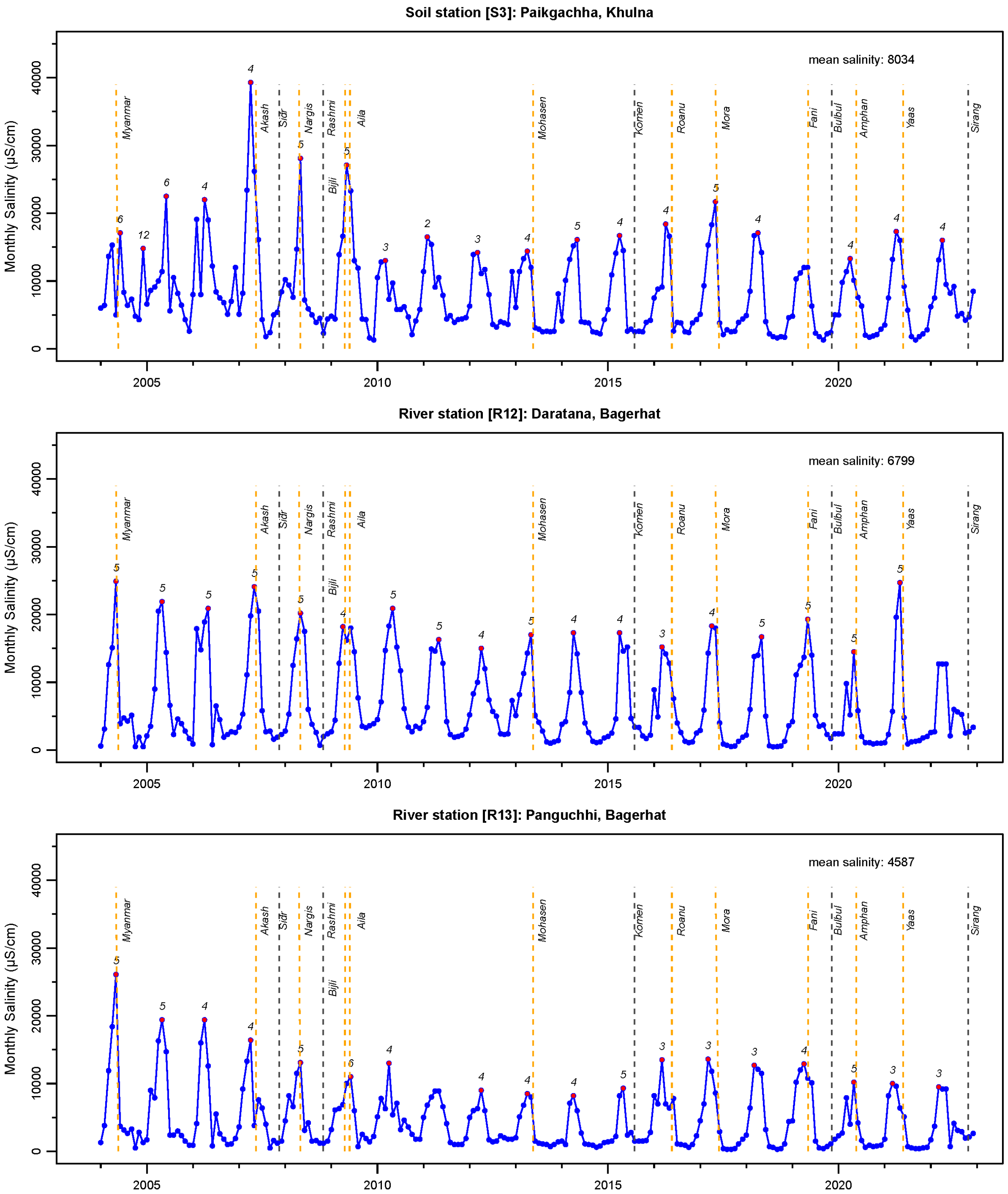

Figure S1. Continued.

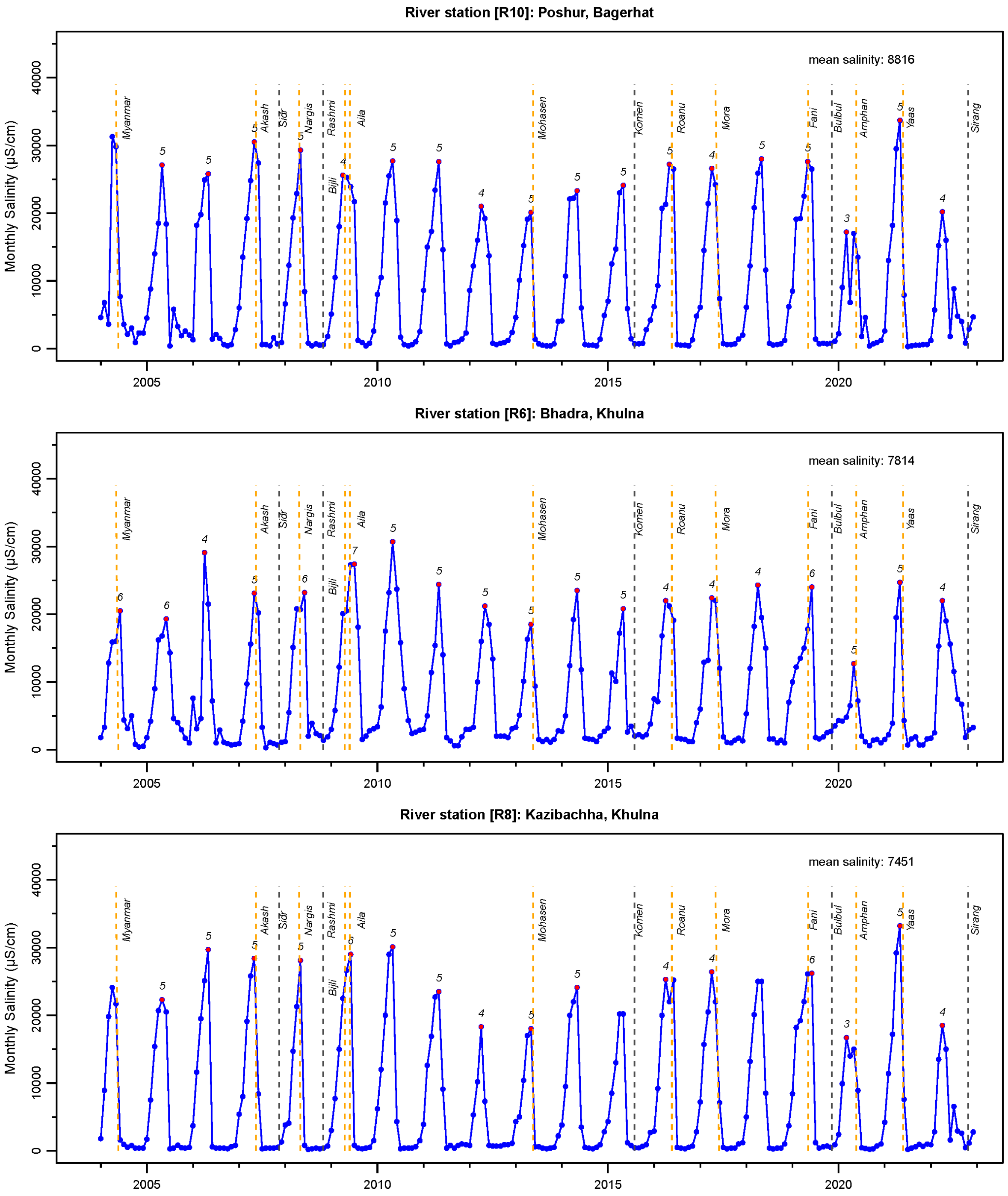

Figure S1. Continued.

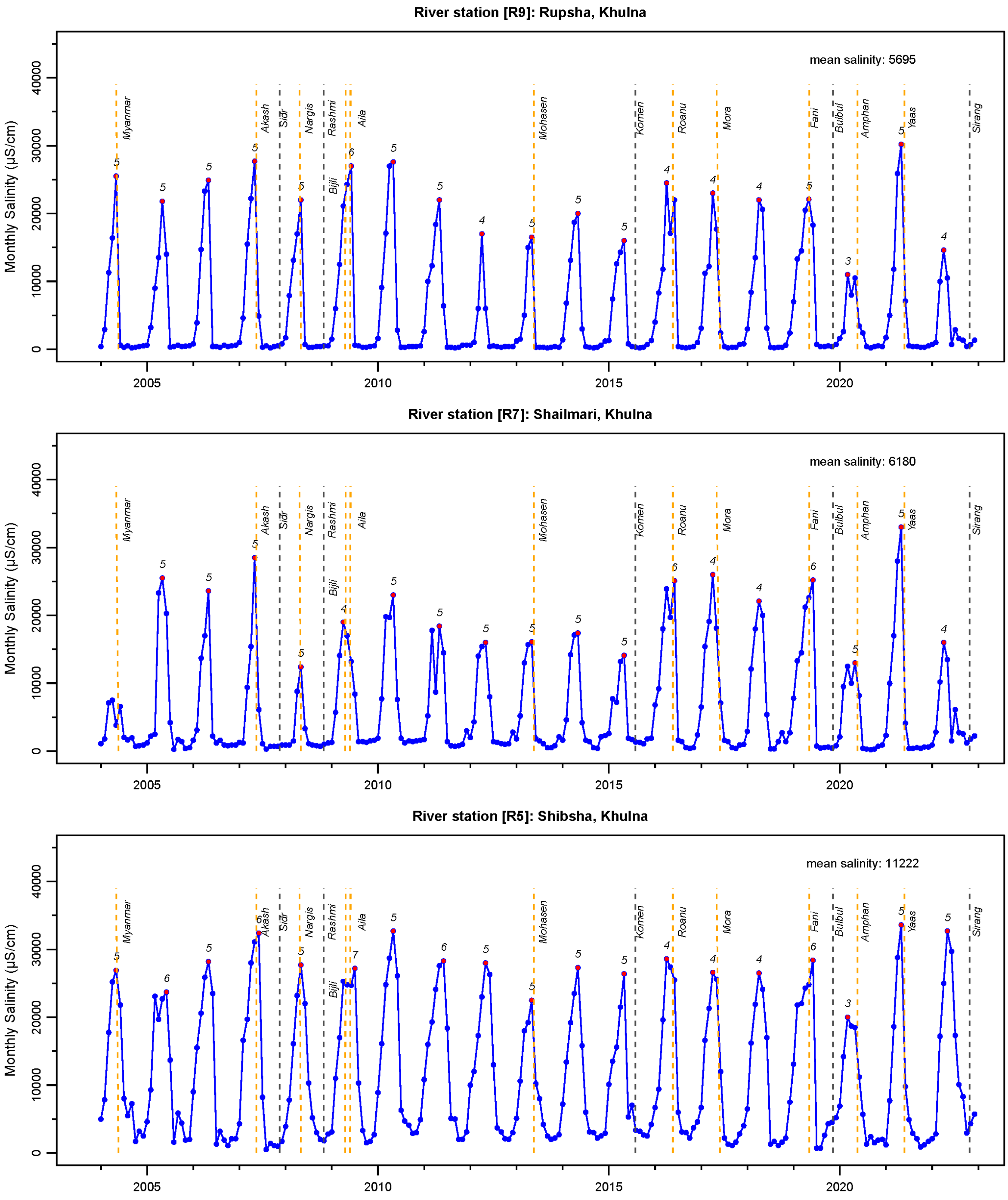

Figure S1. Continued.

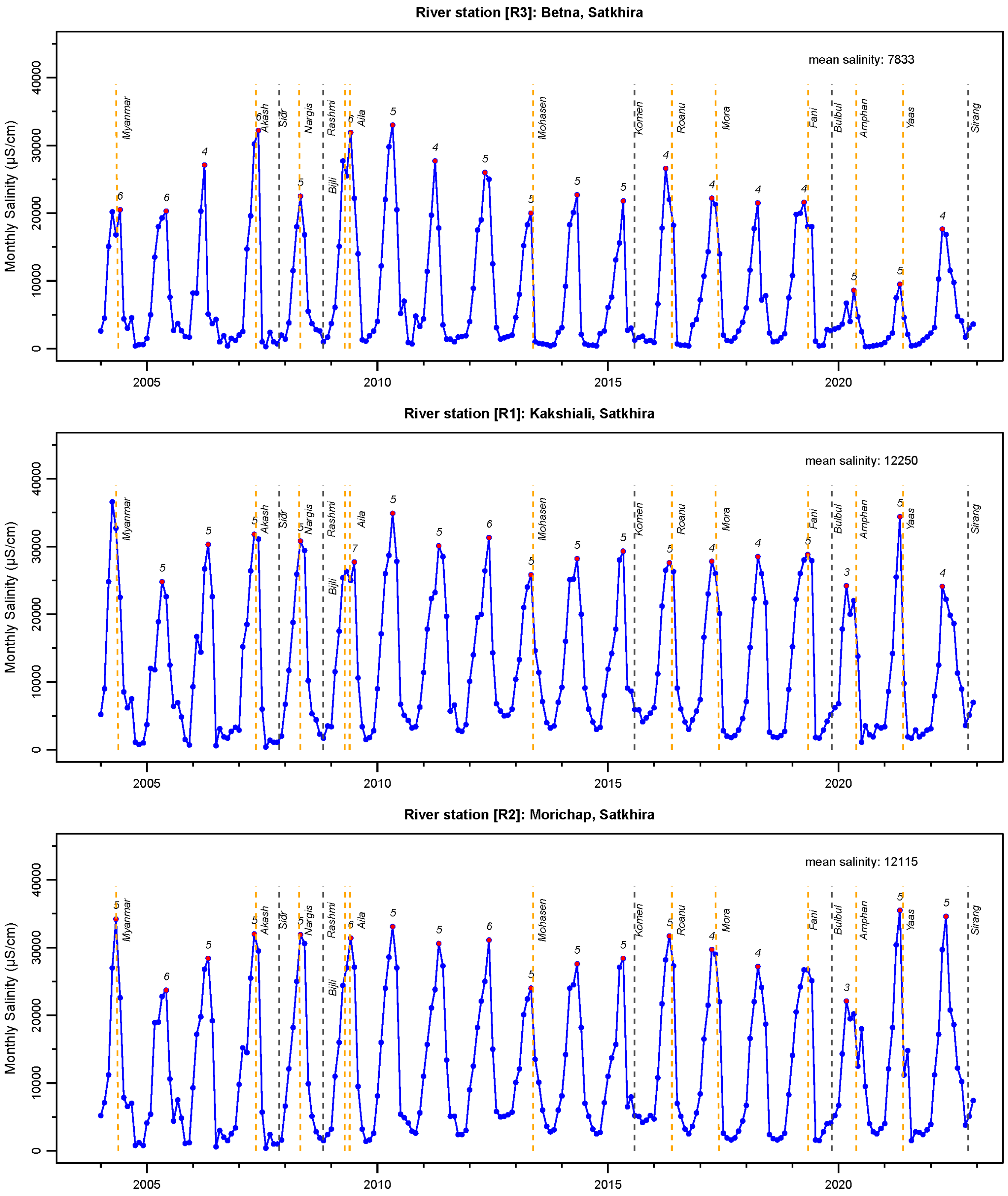

Figure S1. Continued.

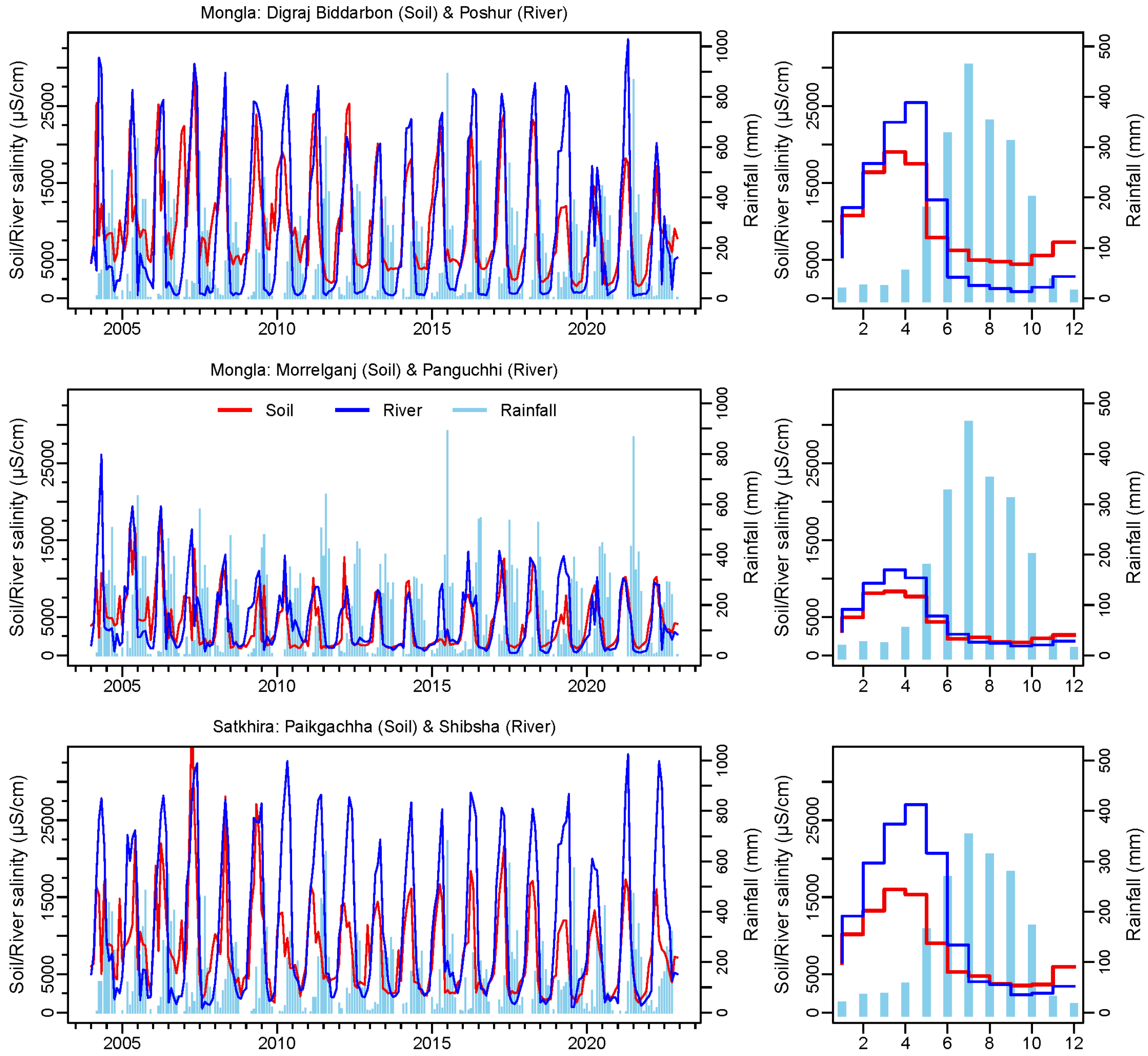

Figure S2. Time-series plots of soil and river water salinity, and rainfall data in southwestern coastal Bangladesh. Time-series (Jan 2004 to Jun 2022) of soil and river water salinity and rainfall at selected stations in southwestern coastal Bangladesh on the left side of the page. The smaller plots on the right-hand side of the page are showing monthly seasonality of salinity and rainfall.

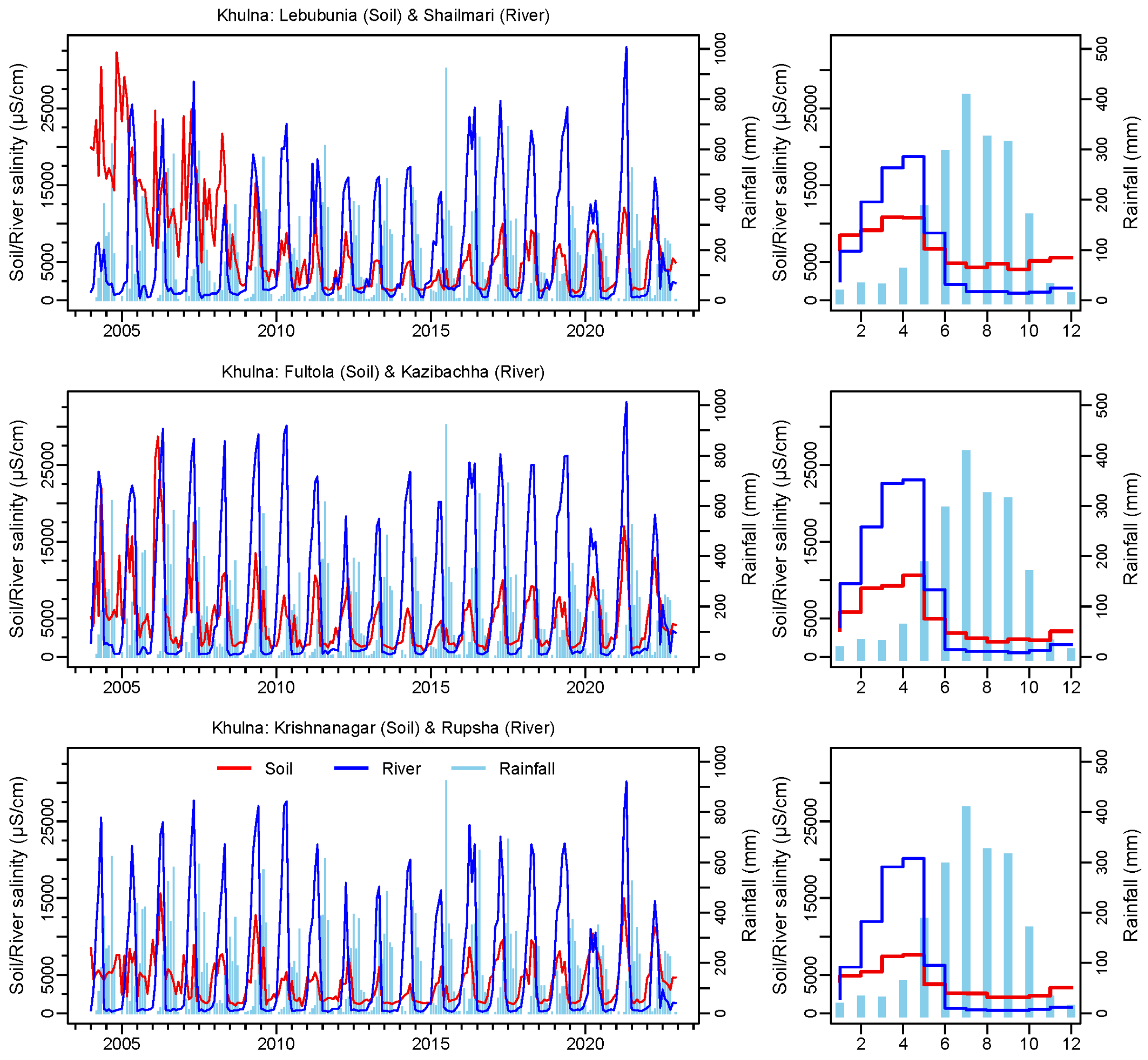

Figure S2. Continued.

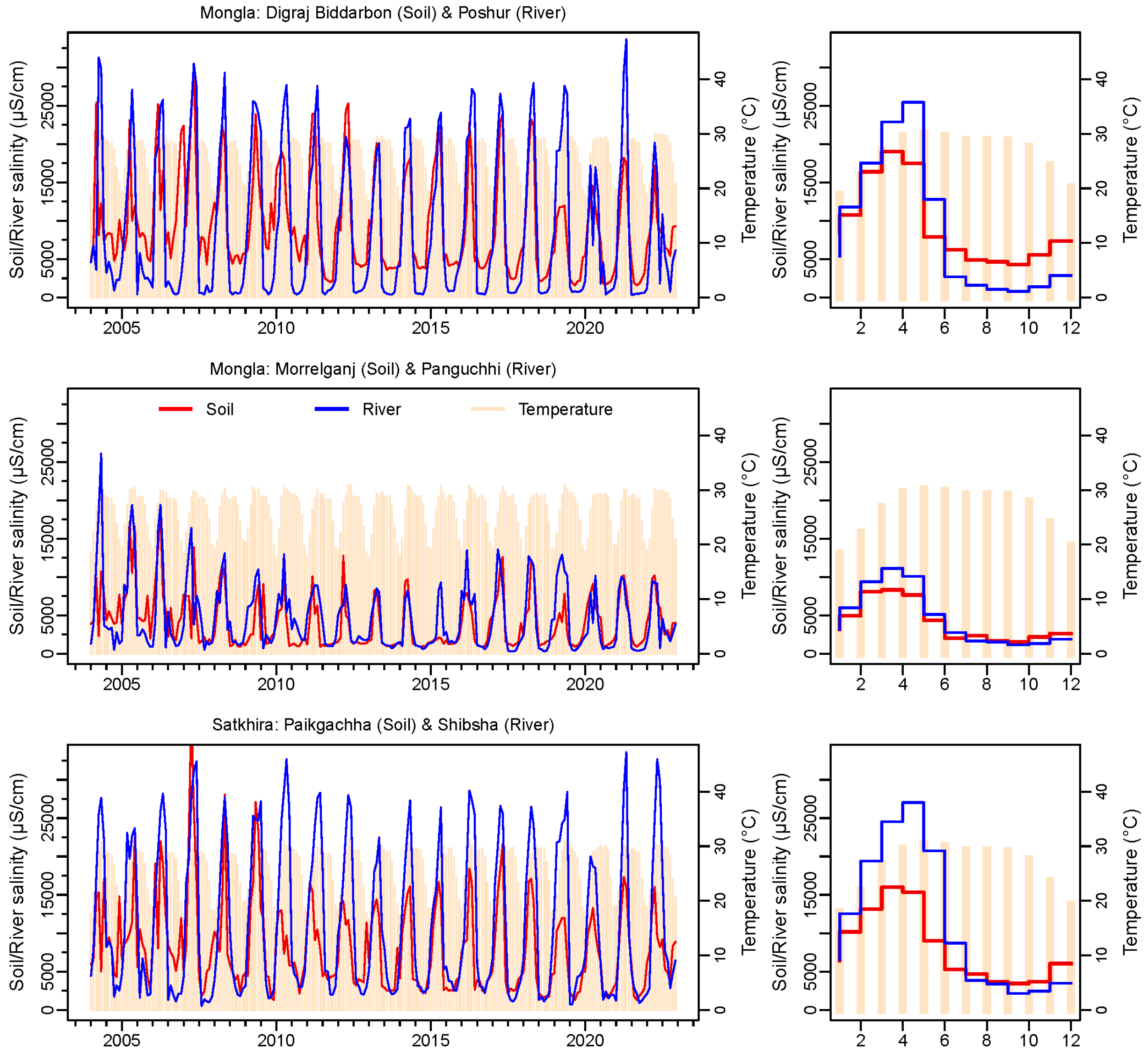

Figure S3. Time-series plots of soil and river water salinity, and temperature data in southwestern coastal Bangladesh. Time-series (Jan 2004 to Jun 2022) of soil and river water salinity and temperature at selected stations in southwestern coastal Bangladesh on the left side of the page. The smaller plots on the right-hand side of the page are showing monthly seasonality of salinity and temperature.

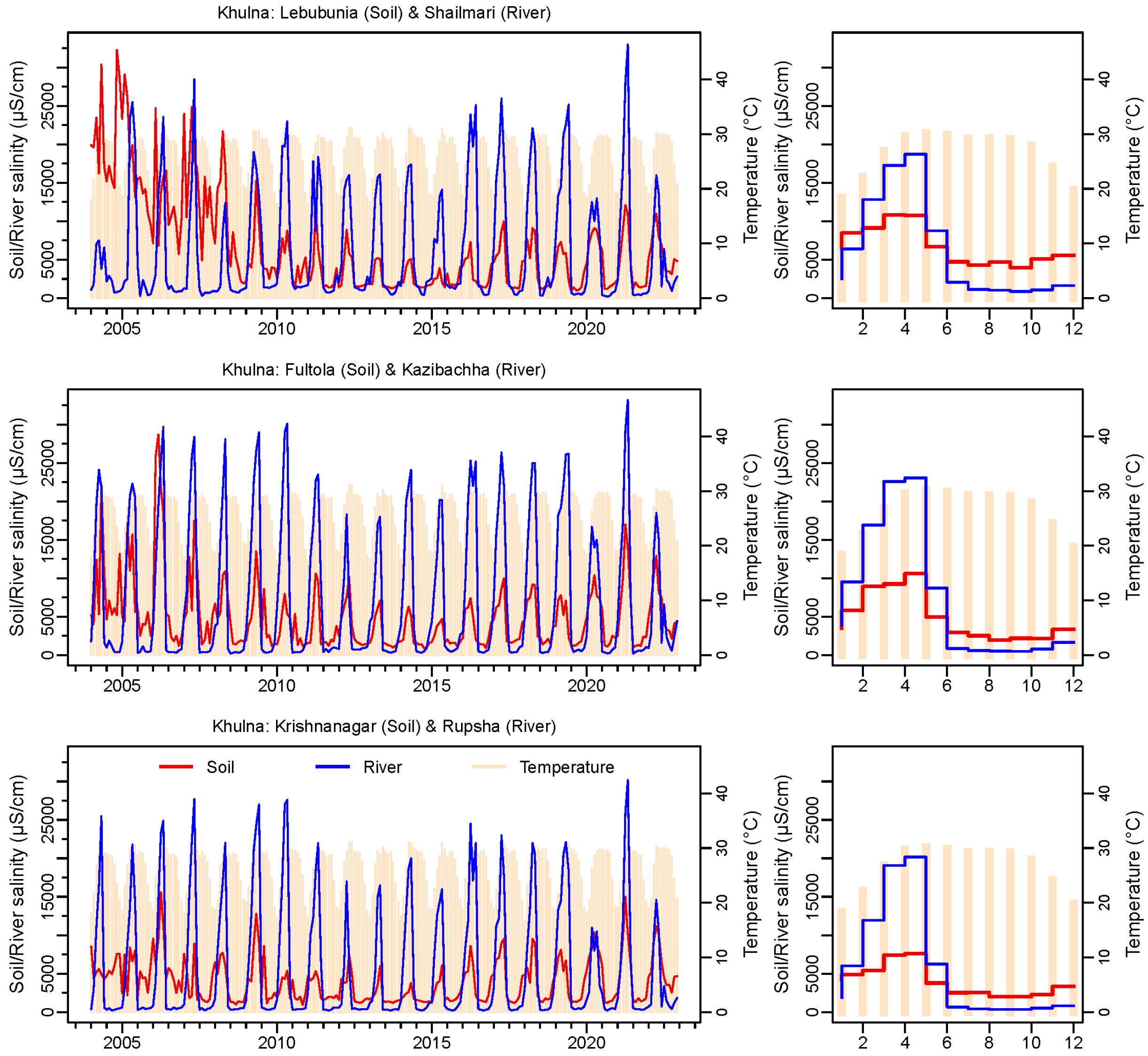

Figure S3. Continued.

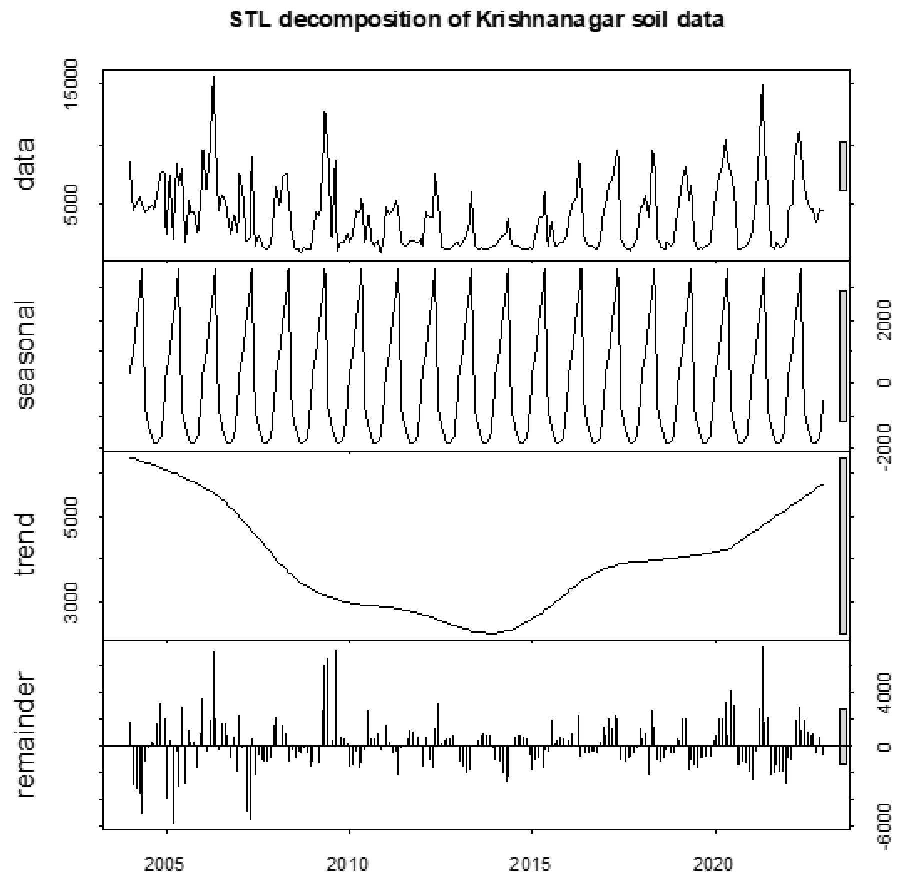

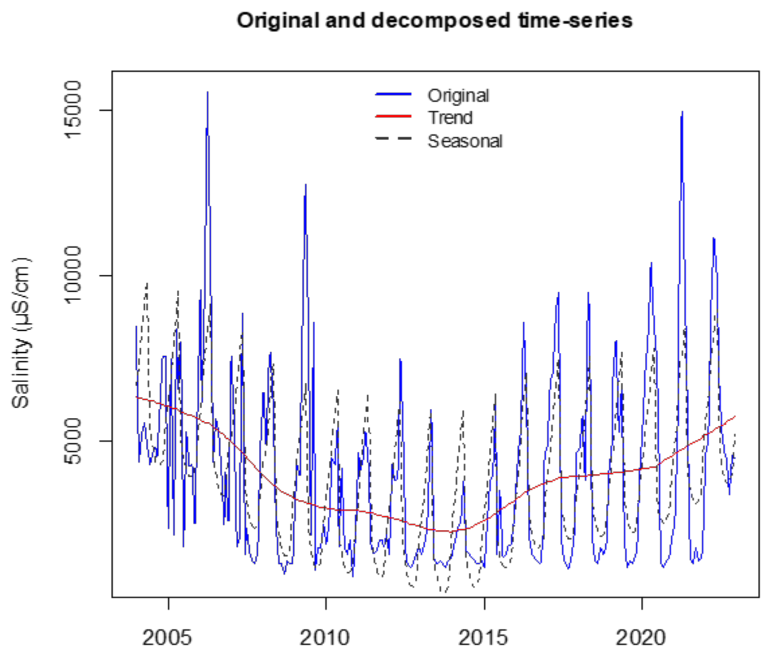

Figure S4. Seasonal-trend decomposition of a soil salinity time-series data. Top panel shows both the original and decomposed time-series data with trend, seasonal and remainder or residual components at Krishnanagar (station ID S5 on Figure 1b) soil salinity data in Khulna. Bottom panel shows the same time-series where the original time-series is plotted with the seasonal and trend components in different colours.

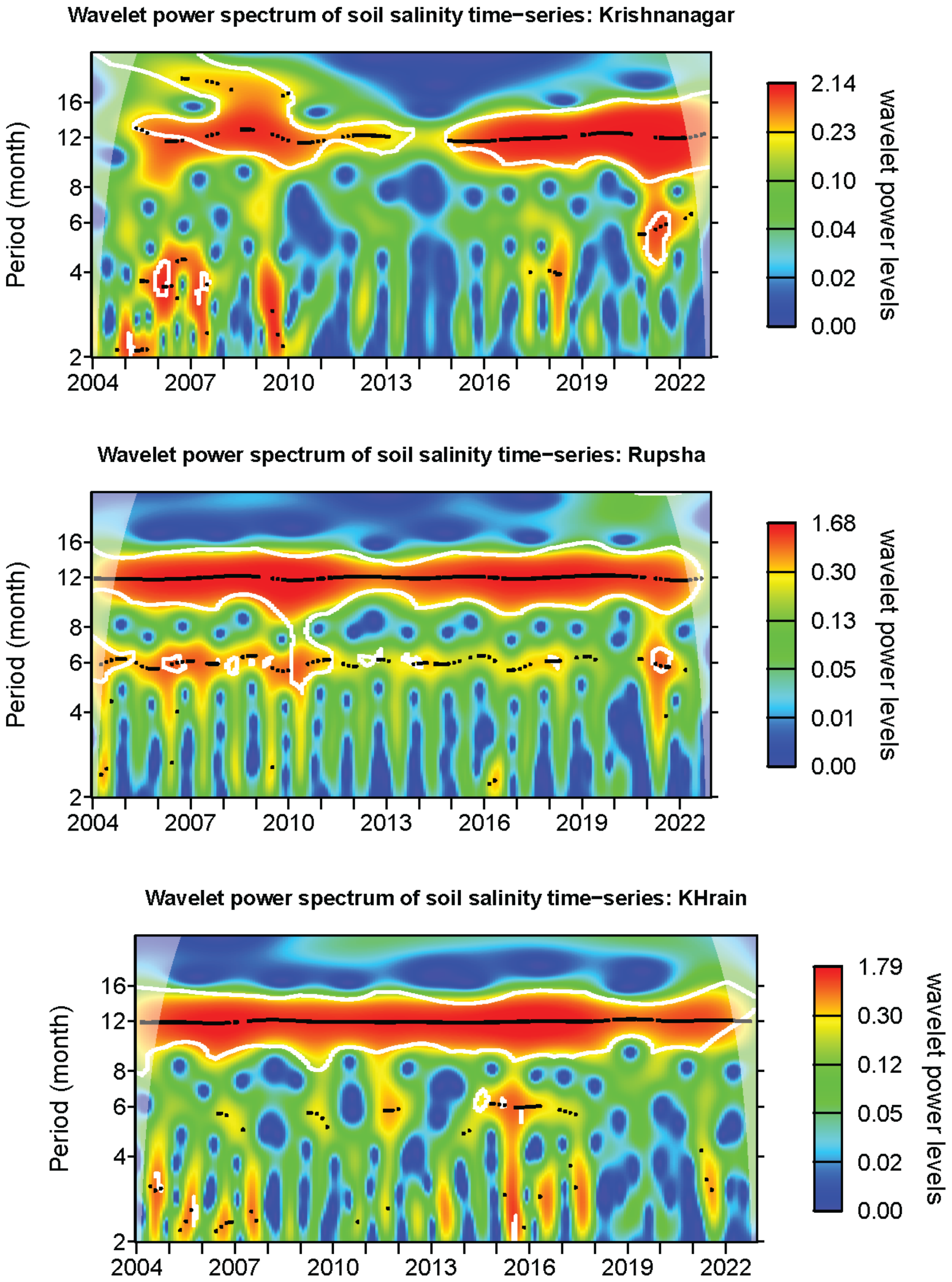

Figure S5. Wavelet analysis of soil salinity time-series data to visualise periodicity. Top panel shows twelve-month periodicity (i.e., seasonality) in soil salinity with the first half of the time-series being not uniform in seasonal patterns. The middle (river water salinity) and bottom (rainfall at Khulna meteorology station) panels show clear annual seasonality in the time-series records all the way from the beginning to the end of the series. There is a half-yearly periodicity in river water salinity suggesting sub-annual fluctuations marking the transition between dry and wet seasons.

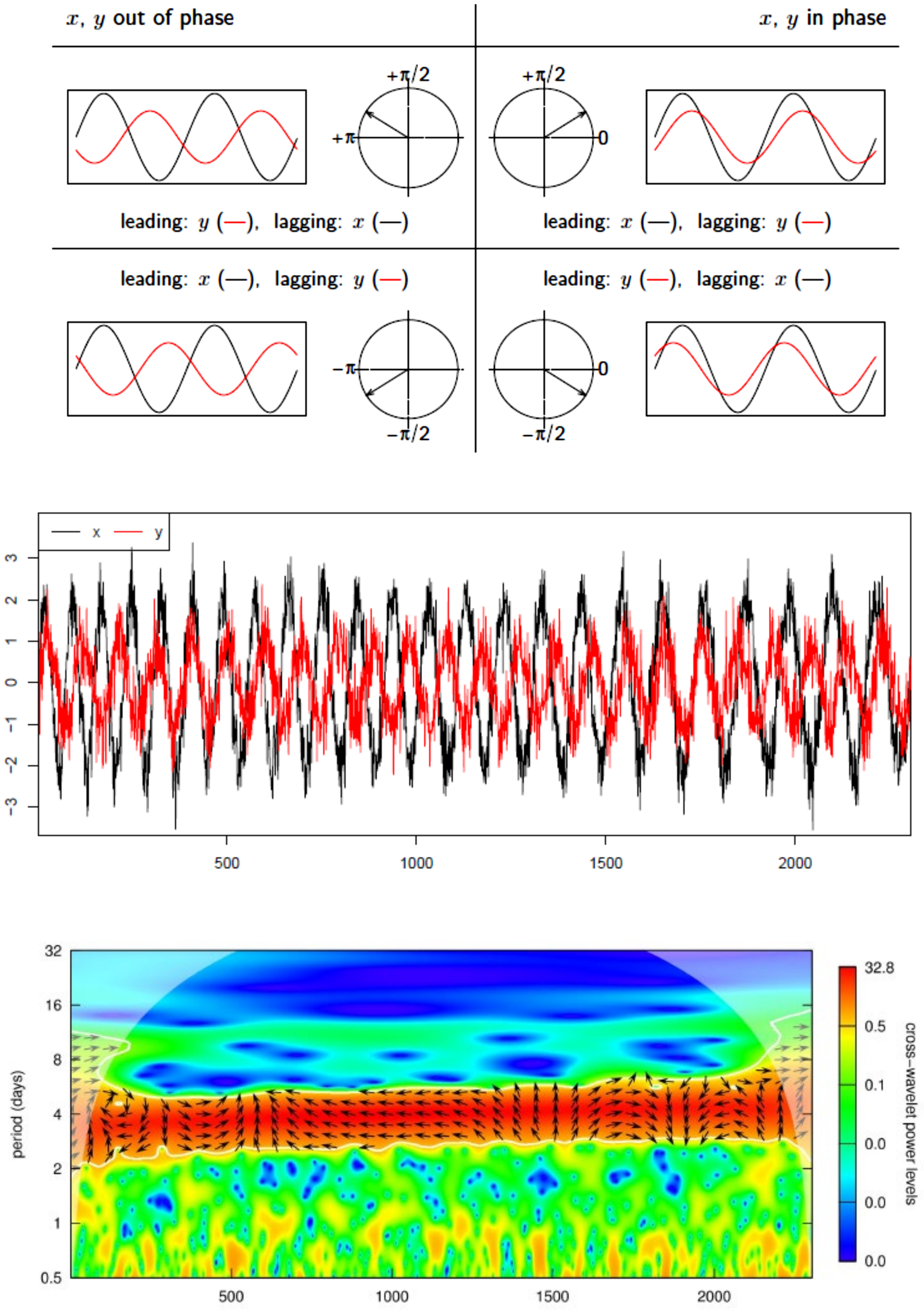

Figure S6. Interpretation of wavelet coherence analysis of time-series data. Top panel shows how to interpret the plots in relation to arrows. In addition to the figure legend, the vertical downward arrows represent negative correlation, while vertical upwards arrows positive correlation between the two time-series data. A zero-phase (i.e., due right or left direction) difference means that the two time series move together on a particular scale. The middle panel shows two hypothetical time-series data, and the bottom panel is the resulting wavelet coherence plots showing how the significant periodicities (e.g., between 2 and 6 days in this example) compared to each other in terms of their correlation along the time.

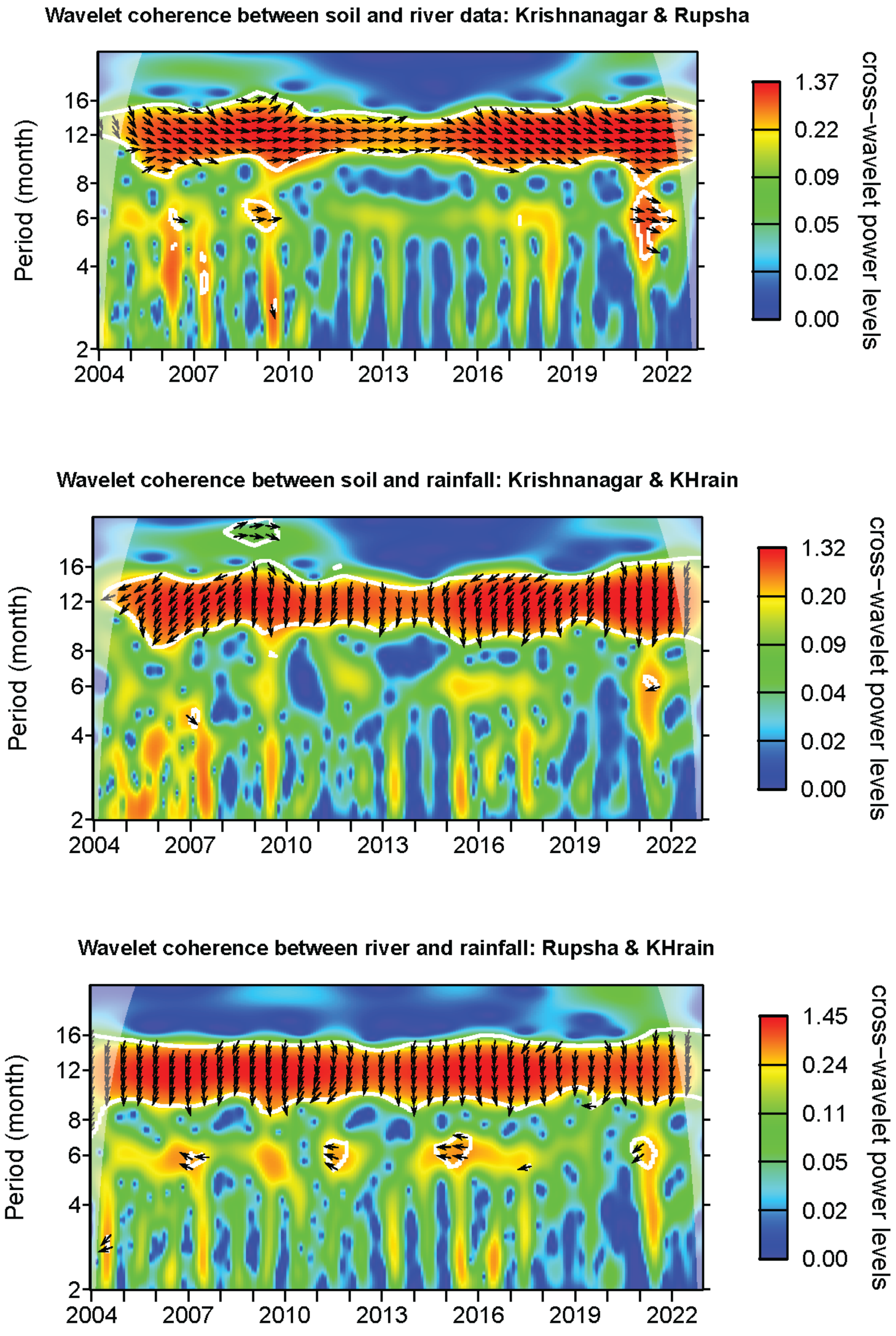

Figure S7. Wavelet coherence analysis of soil, river water salinity and rainfall time-series data to visualise correlation in periodicity. Top panel shows that soil and river water salinity data are correlated, and both are in phase with soil salinity leading river water salinity in some parts of the time-series period. The middle and bottom panels show downward arrows indicating a strong negative correlation between salinity and rainfall with rainfall leading salinity by certain time period.

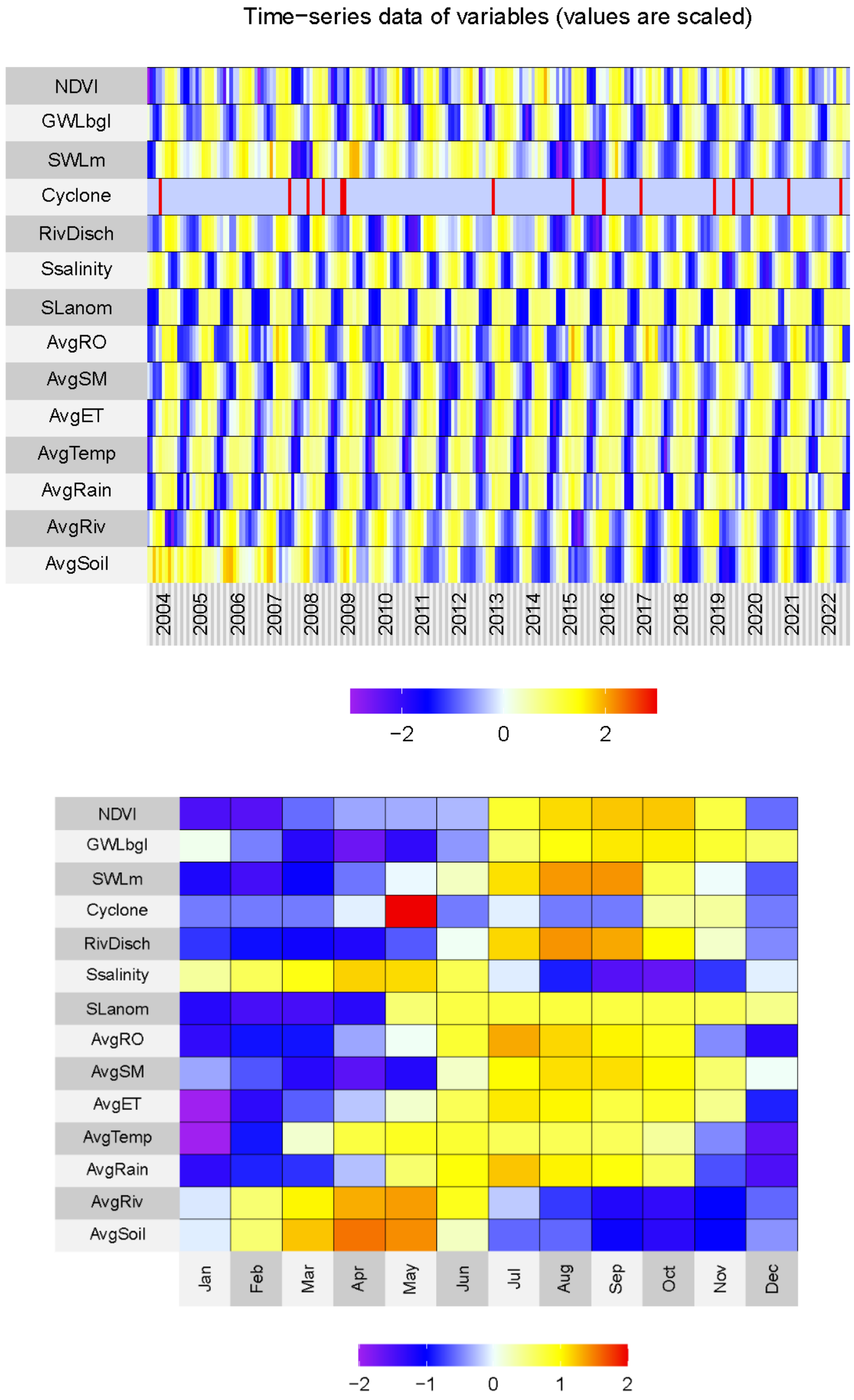

Figure S8. Time-series data of soil and river water salinity, and twelve covariates. Top panel: scaled time-series of soil and river water salinity, and twelve covariate datasets used in the models. **Bottom panel:** scaled seasonality of the same time-series data. All time-series data are monthly. Abbreviations: AvgSoil=mean soil salinity (n=7), AvgRiv=mean surface water (river water) salinity (n=11), AvgRain=mean rainfall (n=4), AvgTemp=mean temperature (n=4), AvgET=mean evapotranspiration (n=4), AvgSM=mean soil moisture (n=4), AvgRO=mean surface runoff (n=4), SLanom=sea-level anomaly, Ssalinity=sea surface salinity, RivDisch=river discharge, Cyclone=timing and number of tropical cyclones, SWLm=surface water levels (m), GWLbgl=groundwater levels (m, below ground level), and NDVI=Normalised Difference Vegetation Index.

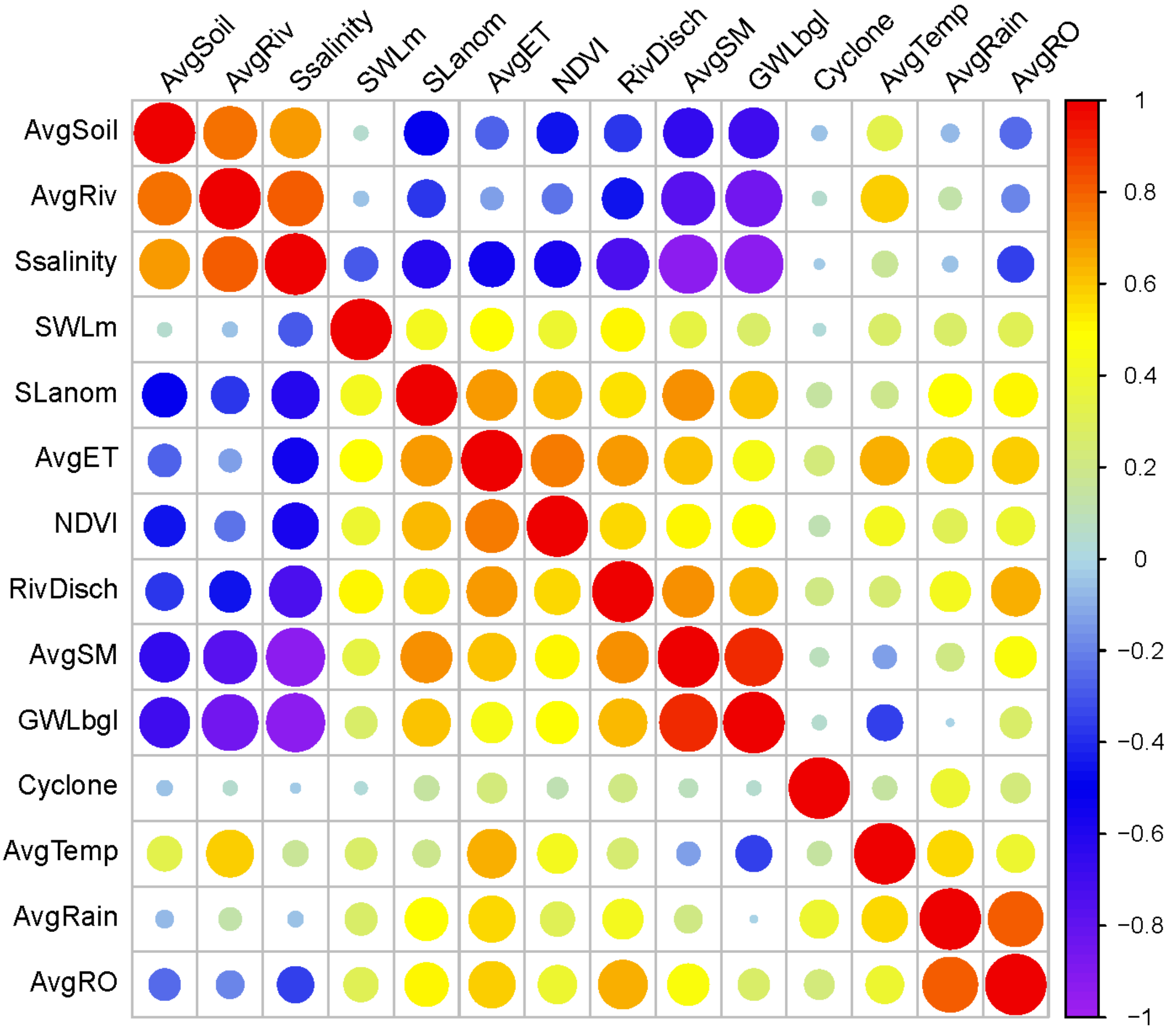

Figure S9. Correlation matrix of time-series data of soil and river water salinity, and twelve covariates. The colour scale on the right-hand side shows Pearson correlation strength and direction. The size of the circles denotes statistical significance (based on the *p* values). Elaboration of the name of the variables is provided in Figure S8.

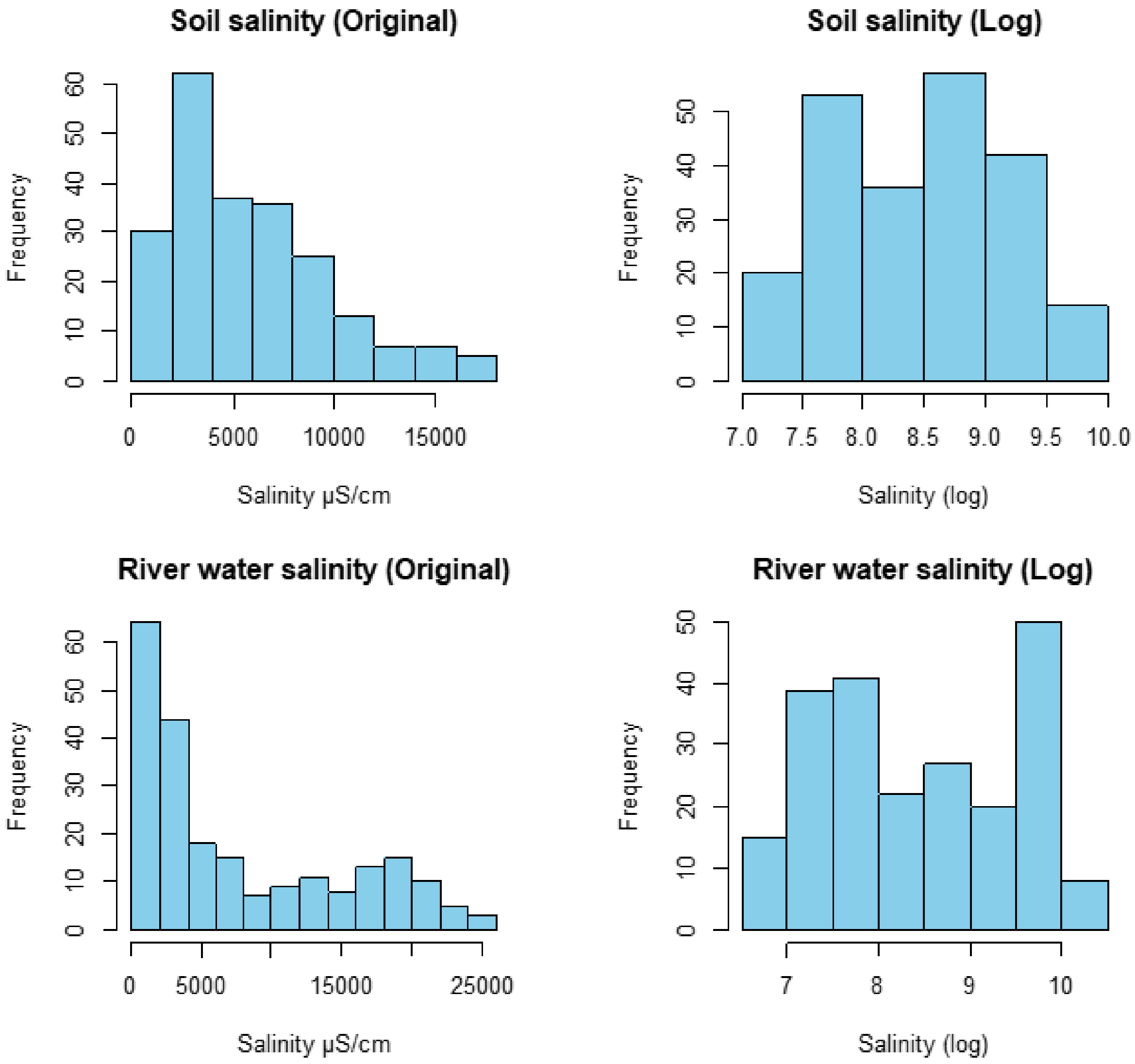

Figure S10. Histograms of soil and river water salinity for original and natural log-transformed values. Soil and river water salinity data duration is 222 months (January 2004 to June 2022). These values are average salinity of 11 soil salinity stations and 13 river water salinity stations in southwestern Bangladesh.

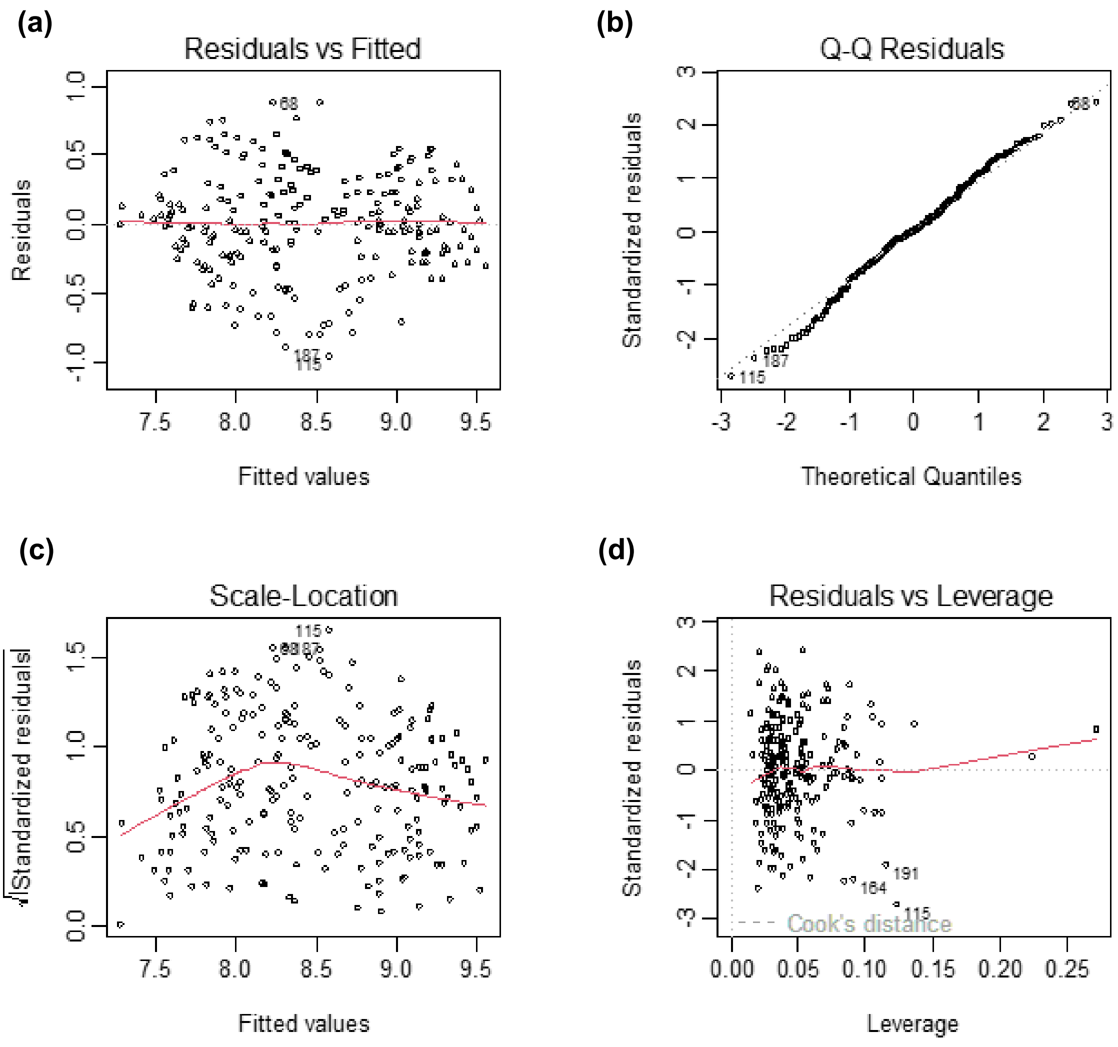

Figure S11. Multiple linear regression model diagnostic plots for soil salinity model with all observations. Diagnostic plots for the linear regression model assessing assumptions of linearity, normality, homoscedasticity, and influential observations. (a) Residuals vs Fitted plot checks for non-linearity and unequal error variances; (b) Normal Q-Q plot assesses whether residuals follow a normal distribution; (c) Scale-Location plot tests for homoscedasticity (constant variance); and (d) Residuals vs Leverage identifies influential observations using Cook's distance.

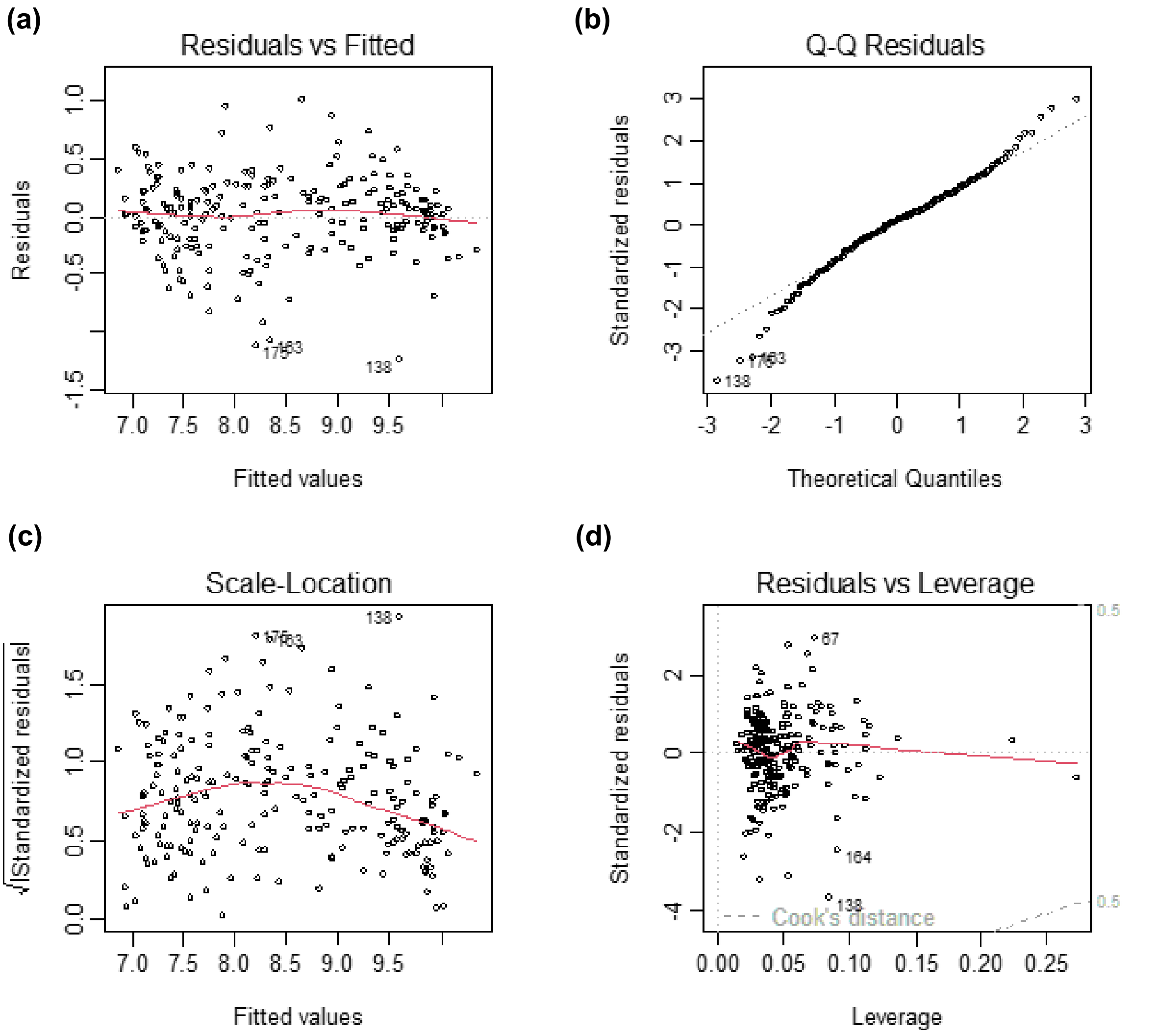

Figure S12. Multiple linear regression model diagnostic plots for river water salinity model with all observations. Diagnostic plots for the linear regression model assessing assumptions of linearity, normality, homoscedasticity, and influential observations. (a) Residuals vs Fitted plot checks for non-linearity and unequal error variances; (b) Normal Q-Q plot assesses whether residuals follow a normal distribution; (c) Scale-Location plot tests for homoscedasticity (constant variance); and (d) Residuals vs Leverage identifies influential observations using Cook's distance.

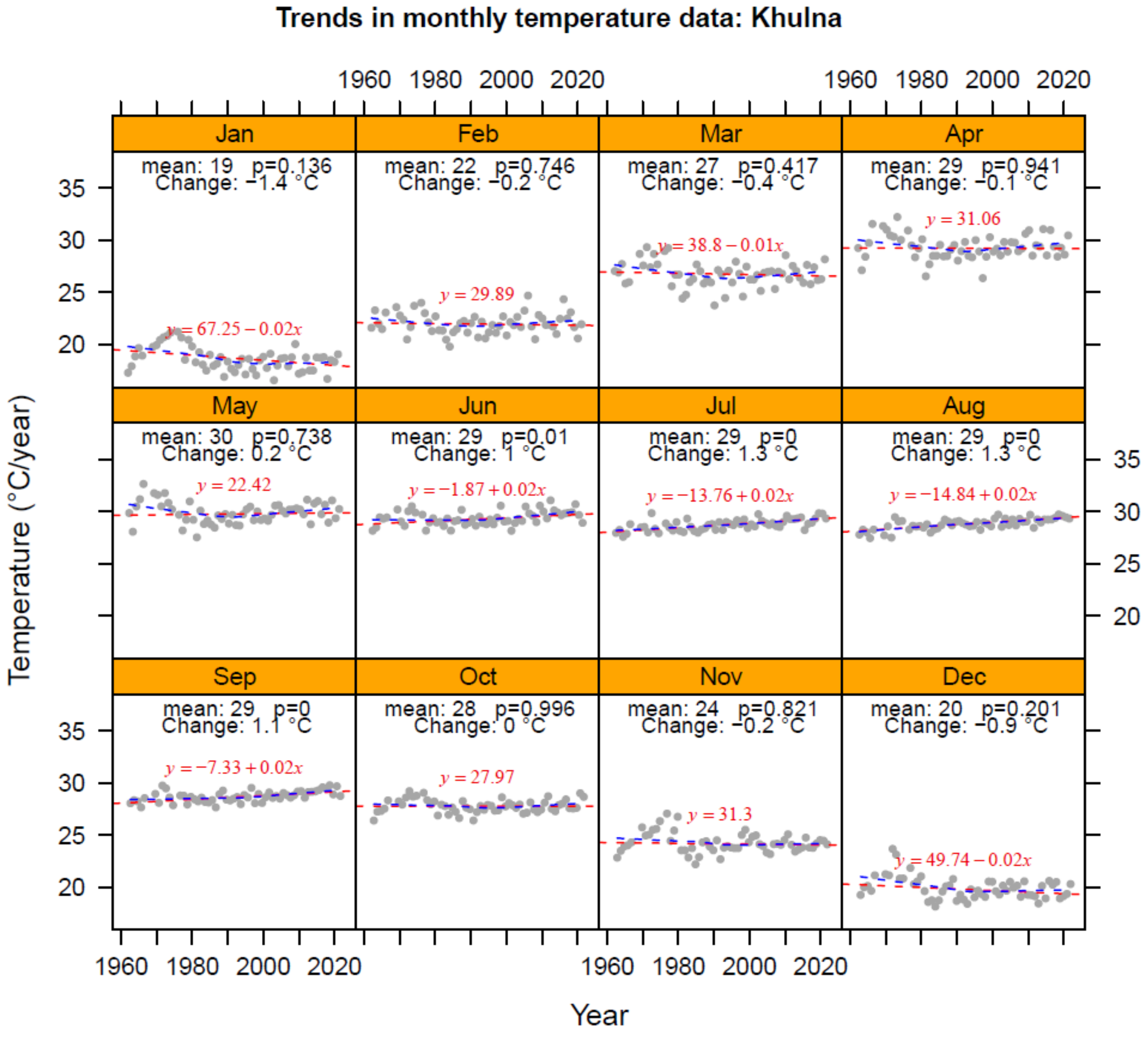

Figure S13. Long-term changes in monthly temperature in coastal Bangladesh. Monthly meteorological data at Khulna station from the Bangladesh Meteorological Department (BMD) are used to calculate linear trends in each calendar month for the period of 1961 to 2022. Long-term temperature is rising in monsoon period (e.g., June to October) but falling in winter and summer months (November to April). Note that not all trends are statistically significant (*p* <0.05 or the trends are statistically significant at the 95% confidence level).

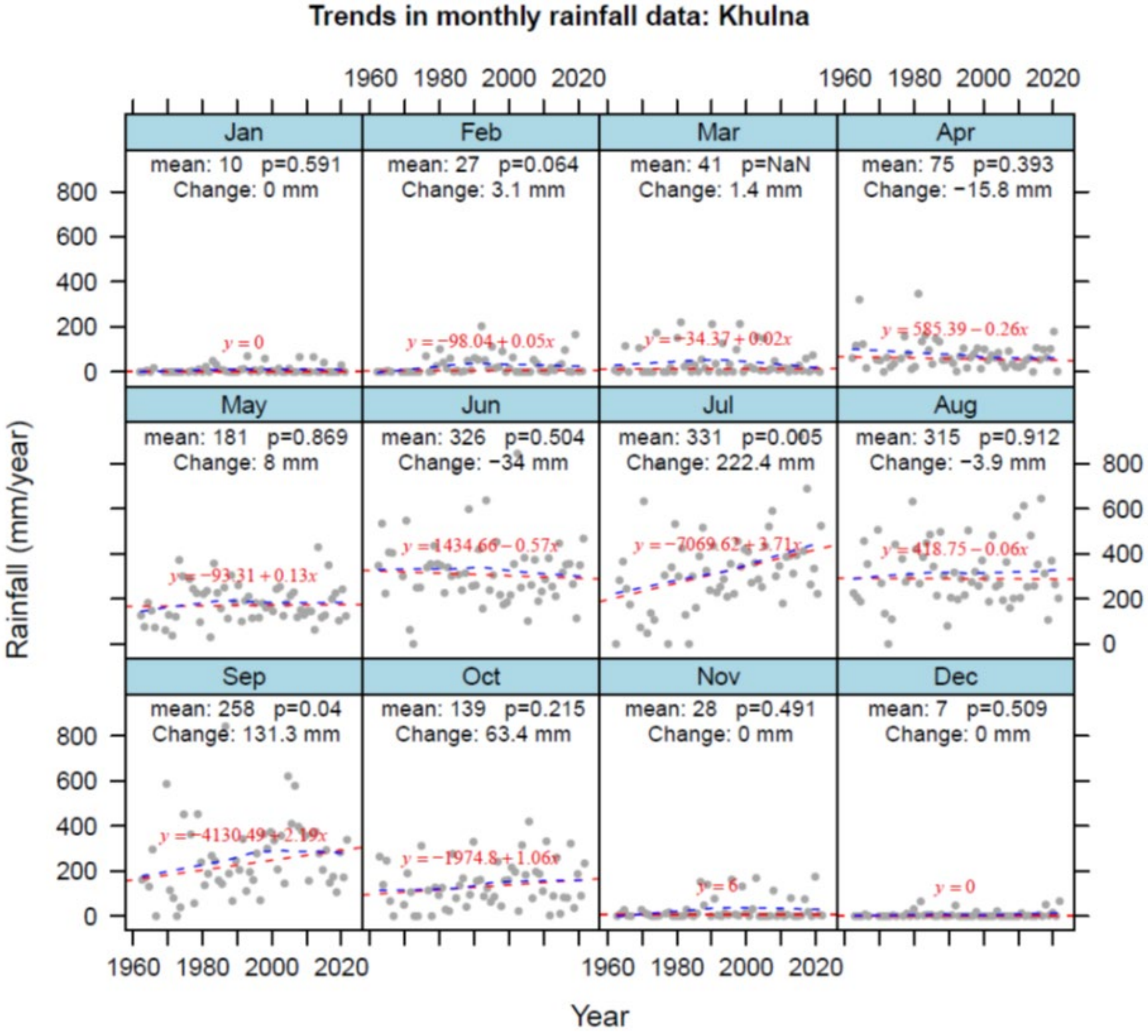

Figure S14. Long-term changes in monthly rainfall in coastal Bangladesh. Monthly meteorological data at Khulna station from the Bangladesh Meteorological Department (BMD) are used to calculate linear trends in each calendar month for the period of 1961 to 2022. Long-term rainfall is rising July, September and October, but falling in April and June. Historically, June has been the month for peak (mean: 326 mm) monsoon rainfall. There is not any change in the winter (November to January) rainfall. Note that not all trends are statistically significant (*p* <0.05 or the trends are statistically significant at the 95% confidence level).

Table S1. Correlation and cross-correlation between river water and soil salinity time-series data with lags (in month). There are 11 soil and 13 river water salinity monitoring stations in the SW coastal Bangladesh. The maximum correlation and corresponding lag in CCF and Pearson and Spearman correlation coefficients are presented here.

River_Station	Soil_Station	Max_correlation	Lag	Pearson	Spearman
Madhumati_river	Morrelganj_soil	1.000	0	1.000	1.000
Poshur_river	Shyamnagar_soil	0.851	0	0.851	0.844
Poshur_river	Koyra_soil	0.851	0	0.851	0.841
Shibsha_river	Shyamnagar_soil	0.849	0	0.849	0.799
Kazibachha_river	Shyamnagar_soil	0.849	0	0.849	0.787
Shibsha_river	Koyra_soil	0.847	0	0.847	0.790
Kazibachha_river	Koyra_soil	0.847	0	0.847	0.787
Morichap_river	Koyra_soil	0.840	0	0.840	0.788
Morichap_river	Shyamnagar_soil	0.839	0	0.839	0.794
Kazibachha_river	Sarankhola_soil	0.837	0	0.837	0.760
Madhumati_river	Dakop_soil	0.834	0	0.834	0.883
Madhumati_river	Sarankhola_soil	0.832	0	0.832	0.854
Poshur_river	Sarankhola_soil	0.830	0	0.830	0.821
Kazibachha_river	Dakop_soil	0.826	0	0.826	0.811
Poshur_river	Dakop_soil	0.821	0	0.821	0.844
Rupsha_river	Shyamnagar_soil	0.820	0	0.820	0.814
Rupsha_river	Koyra_soil	0.818	0	0.818	0.818
Morichap_river	Dakop_soil	0.815	1	0.797	0.773
Kakshiali_river	Koyra_soil	0.813	0	0.813	0.753
Daratana_river	Shyamnagar_soil	0.813	0	0.813	0.762
Daratana_river	Koyra_soil	0.813	0	0.813	0.758
Kakshiali_river	Shyamnagar_soil	0.812	0	0.812	0.759
Rupsha_river	Sarankhola_soil	0.811	0	0.811	0.787
Shibsha_river	Dakop_soil	0.811	0	0.811	0.784
Madhumati_river	Shyamnagar_soil	0.810	0	0.810	0.846
Daratana_river	Sarankhola_soil	0.808	0	0.808	0.755
Shibsha_river	Sarankhola_soil	0.806	0	0.806	0.766
Morichap_river	Sarankhola_soil	0.799	1	0.793	0.753
Madhumati_river	Koyra_soil	0.796	0	0.796	0.841
Rupsha_river	Dakop_soil	0.790	0	0.790	0.824
Shailmari_river	Shyamnagar_soil	0.789	0	0.789	0.772
Daratana_river	Dakop_soil	0.786	0	0.786	0.748
Kakshiali_river	Dakop_soil	0.785	1	0.778	0.751
Panguchhi_river	Shyamnagar_soil	0.785	0	0.785	0.811
Panguchhi_river	Koyra_soil	0.784	0	0.784	0.803
Betna_river	Koyra_soil	0.781	0	0.781	0.788
Betna_river	Shyamnagar_soil	0.780	0	0.780	0.787
Shailmari_river	Koyra_soil	0.779	0	0.779	0.768
Bhadra_river	Koyra_soil	0.776	0	0.776	0.750
Bhadra_river	Shyamnagar_soil	0.775	1	0.774	0.753
Kakshiali_river	Sarankhola_soil	0.774	0	0.774	0.732
Shailmari_river	Sarankhola_soil	0.771	0	0.771	0.759
Madhumati_river	Fultola_soil	0.771	0	0.771	0.838
Kapotaksha_river	Koyra_soil	0.765	0	0.765	0.731
Kapotaksha_river	Shyamnagar_soil	0.762	0	0.762	0.728

Panguchhi_river	Dakop_soil	0.761	0	0.761	0.806
Panguchhi_river	Sarankhola_soil	0.760	0	0.760	0.797
Bhadra_river	Dakop_soil	0.759	1	0.738	0.748
Morichap_river	Digraj_Biddarbon_soil	0.758	1	0.677	0.688
Kapotaksha_river	Dakop_soil	0.755	0	0.755	0.750
Bhadra_river	Sarankhola_soil	0.753	1	0.749	0.729
Poshur_river	Paikgachha_soil	0.753	0	0.753	0.790
Shailmari_river	Dakop_soil	0.751	0	0.751	0.775
Kapotaksha_river	Sarankhola_soil	0.749	0	0.749	0.706
Kazibachha_river	Paikgachha_soil	0.748	0	0.748	0.732
Madhumati_river	Krishnanagar_soil	0.745	0	0.745	0.814
Kakshiali_river	Digraj_Biddarbon_soil	0.743	1	0.673	0.676
Kazibachha_river	Digraj_Biddarbon_soil	0.742	0	0.742	0.720
Betna_river	Sarankhola_soil	0.740	0	0.740	0.763
Poshur_river	Digraj_Biddarbon_soil	0.736	0	0.736	0.757
Shibsha_river	Digraj_Biddarbon_soil	0.735	1	0.693	0.708
Betna_river	Dakop_soil	0.732	0	0.732	0.791
Shibsha_river	Paikgachha_soil	0.728	0	0.728	0.757
Morichap_river	Paikgachha_soil	0.727	1	0.710	0.738
Bhadra_river	Digraj_Biddarbon_soil	0.727	1	0.633	0.682
Rupsha_river	Paikgachha_soil	0.721	0	0.721	0.740
Daratana_river	Paikgachha_soil	0.719	0	0.719	0.710
Daratana_river	Digraj_Biddarbon_soil	0.719	0	0.719	0.736
Rupsha_river	Digraj_Biddarbon_soil	0.714	0	0.714	0.756
Kakshiali_river	Paikgachha_soil	0.712	0	0.712	0.735
Betna_river	Digraj_Biddarbon_soil	0.710	1	0.706	0.754
Betna_river	Paikgachha_soil	0.704	0	0.704	0.762
Panguchhi_river	Kismat_Fultola_soil	0.692	0	0.692	0.714
Panguchhi_river	Morrelganj_soil	0.691	0	0.691	0.694
Madhumati_river	Paikgachha_soil	0.686	-12	0.651	0.755
Shailmari_river	Digraj_Biddarbon_soil	0.683	0	0.683	0.730
Kazibachha_river	Kismat_Fultola_soil	0.683	0	0.683	0.700
Kazibachha_river	Morrelganj_soil	0.683	0	0.683	0.701
Bhadra_river	Paikgachha_soil	0.681	1	0.672	0.712
Rupsha_river	Kismat_Fultola_soil	0.679	0	0.679	0.702
Madhumati_river	Digraj_Biddarbon_soil	0.679	0	0.679	0.769
Shailmari_river	Paikgachha_soil	0.677	0	0.677	0.738
Madhumati_river	Kismat_Fultola_soil	0.677	0	0.677	0.744
Daratana_river	Kismat_Fultola_soil	0.674	0	0.674	0.656
Daratana_river	Fultola_soil	0.672	0	0.672	0.629
Bhadra_river	Kismat_Fultola_soil	0.671	1	0.616	0.695
Panguchhi_river	Fultola_soil	0.671	0	0.671	0.708
Kapotaksha_river	Kismat_Fultola_soil	0.669	1	0.656	0.708
Morichap_river	Kismat_Fultola_soil	0.658	1	0.627	0.705
Shibsha_river	Morrelganj_soil	0.657	1	0.611	0.622
Panguchhi_river	Paikgachha_soil	0.656	0	0.656	0.757
Morichap_river	Morrelganj_soil	0.647	1	0.605	0.613
Rupsha_river	Morrelganj_soil	0.645	0	0.645	0.719
Shibsha_river	Kismat_Fultola_soil	0.643	0	0.643	0.724
Bhadra_river	Morrelganj_soil	0.643	1	0.580	0.613
Poshur_river	Morrelganj_soil	0.643	0	0.643	0.722
Kapotaksha_river	Morrelganj_soil	0.642	1	0.620	0.644
Kazibachha_river	Fultola_soil	0.642	0	0.642	0.668

Poshur_river	Fultola_soil	0.641	0	0.641	0.724
Kakshiali_river	Kismat_Fultola_soil	0.640	1	0.604	0.678
Kapotaksha_river	Paikgachha_soil	0.639	0	0.639	0.660
Kapotaksha_river	Digraj_Biddarbon_soil	0.639	1	0.574	0.609
Daratana_river	Morrelganj_soil	0.639	0	0.639	0.593
Shailmari_river	Morrelganj_soil	0.637	0	0.637	0.652
Morichap_river	Krishnanagar_soil	0.631	1	0.567	0.568
Kazibachha_river	Krishnanagar_soil	0.631	0	0.631	0.656
Rupsha_river	Fultola_soil	0.629	0	0.629	0.691
Poshur_river	Kismat_Fultola_soil	0.629	0	0.629	0.730
Shibsha_river	Fultola_soil	0.621	0	0.621	0.675
Panguchhi_river	Digraj_Biddarbon_soil	0.621	0	0.621	0.750
Kapotaksha_river	Fultola_soil	0.619	1	0.619	0.699
Kakshiali_river	Morrelganj_soil	0.618	1	0.571	0.574
Shibsha_river	Krishnanagar_soil	0.616	1	0.569	0.594
Rupsha_river	Krishnanagar_soil	0.611	0	0.611	0.676
Betna_river	Kismat_Fultola_soil	0.610	0	0.610	0.722
Morichap_river	Fultola_soil	0.606	1	0.600	0.641
Poshur_river	Krishnanagar_soil	0.603	-12	0.595	0.685
Shailmari_river	Krishnanagar_soil	0.601	0	0.601	0.601
Bhadra_river	Fultola_soil	0.583	1	0.533	0.646
Betna_river	Morrelganj_soil	0.577	0	0.577	0.661
Kakshiali_river	Krishnanagar_soil	0.572	1	0.517	0.528
Shailmari_river	Kismat_Fultola_soil	0.571	0	0.571	0.705
Kakshiali_river	Fultola_soil	0.566	1	0.559	0.617
Panguchhi_river	Krishnanagar_soil	0.566	0	0.566	0.645
Bhadra_river	Krishnanagar_soil	0.560	1	0.543	0.565
Betna_river	Fultola_soil	0.556	0	0.556	0.698
Kapotaksha_river	Krishnanagar_soil	0.552	0	0.552	0.576
Shailmari_river	Fultola_soil	0.549	0	0.549	0.654
Madhumati_river	Lebubunia_soil	0.545	0	0.545	0.756
Daratana_river	Krishnanagar_soil	0.540	-12	0.539	0.521
Betna_river	Krishnanagar_soil	0.482	0	0.482	0.591
Panguchhi_river	Lebubunia_soil	0.449	0	0.449	0.586
Kapotaksha_river	Lebubunia_soil	0.448	1	0.418	0.503
Daratana_river	Lebubunia_soil	0.379	12	0.361	0.462
Kazibachha_river	Lebubunia_soil	0.346	-12	0.335	0.467
Shibsha_river	Lebubunia_soil	0.345	1	0.321	0.465
Poshur_river	Lebubunia_soil	0.340	0	0.340	0.539
Rupsha_river	Lebubunia_soil	0.321	-12	0.321	0.510
Kakshiali_river	Lebubunia_soil	0.319	-11	0.278	0.390
Morichap_river	Lebubunia_soil	-0.310	7	0.272	0.401
Bhadra_river	Lebubunia_soil	0.296	1	0.252	0.438
Betna_river	Lebubunia_soil	0.295	1	0.271	0.484
Shailmari_river	Lebubunia_soil	-0.265	-5	0.195	0.432

Table S2. Descriptive statistics, trends and seasonal-trend decomposition of soil salinity data. There are 11 soil salinity monitoring stations in the SW coastal Bangladesh.

StationName	Morrelganj	Sarankhola	Digraj_Biddarbon	Fultola	Lebubunia	Koyra
StationID	S10	S11	S9	S7	S4	S2
Parameter	Soil water	Soil water	Soil water	Soil water	Soil water	Soil water
District	Bagerhat	Bagerhat	Bagerhat	Khulna	Khulna	Khulna
Upazila	Morrelganj	Sarankhola	Mongla	Batiaghta	Dumuria	Koyra
Union	Sadar	Dhansagar	Burirdanga	Batiaghata	Gutudia	Islampur
Village	NA	Dhansagar	Digraj Biddarbahon	Fultala	Lebubunia	Bamiya
DecLat	22.4699	22.3855	22.5371	22.7085	22.7879	22.4508
DecLon	89.8643	89.8124	89.5926	89.5288	89.483	89.2833
StnName	Morrelganj	Sarankhola	Digraj_Biddarbon	Fultola	Lebubunia	Koyra
StnType	Soil water	Soil water	Soil water	Soil water	Soil water	Soil water
ECAvgMin	900	1100	1600	900	900	1000
ECAvgMax	18600	12000	30400	28700	32300	12500
ECAvgLTMed	2400	2978	7896	3800	4550	3808
ECAvgLTavg	4097	3949	9420	4859	6765	4544
ECAvgLTsd	3526	2385	6384	4454	6533	2398
ECAvgDRYavg	5288	4815	12132	6217	8071	5421
ECAvgWETavg	2430	2737	5625	2957	4936	3316
ECavgLinTD	-110.74	-66.99	-253.31	-180.16	-702.13	-62.70
ECavgLNTDerr	42.03	28.52	75.40	52.55	63.79	28.71
ECavgLNTDp	0.01	0.02	0.00	0.00	0.00	0.03
ECavgSenTD	-44.86	-78.46	-229.60	-60.71	-448.98	-79.04
ECavgSenTDp	0.02	0.00	0.00	0.01	0.00	0.00
ECavgSSenTD	-28.57	-65.25	-244.44	-44.95	-360.00	-55.29
ECavgSTLTD	-99.27	0.22	2.91	-152.09	-825.71	0.57
ECavgMKTau	-0.10	-0.17	-0.18	-0.11	-0.36	-0.15
ECavgSMKTau	-0.13	-0.27	-0.31	-0.13	-0.37	-0.24
ECavgDryTD	-86.37	-38.08	-239.27	-192.87	-736.18	-35.95
ECavgDryTDerr	58.68	39.15	105.95	78.81	93.31	38.50
ECavgDryTDp	0.14	0.33	0.03	0.02	0.00	0.35
ECavgWetTD	-133.36	-99.17	-246.53	-149.13	-642.00	-91.72
ECavgWetTDerr	42.65	28.45	48.11	40.88	68.10	30.86
ECavgWetTDp	0.00	0.00	0.00	0.00	0.00	0.00
ECavgSTLt	12.45	6.19	4.46	18.31	67.60	7.45
ECavgSTLs	51.20	78.40	68.47	45.85	11.91	81.90
ECavgSTLr	36.35	15.41	27.07	35.84	20.49	10.65

StationName	Koyra	Dakop	Kismat_Fultola	Krishnanagar	Paikgachha	Shyamnagar
StationID	S2	S8	S6	S5	S3	S1
Parameter	Soil water	Soil water	Soil water	Soil water	Soil water	Soil water
District	Khulna	Khulna	Khulna	Khulna	Khulna	Satkhira
Upazila	Koyra	Dakop	Batiaghta	Batiaghta	Paikgachha	Shyamnagar
Union	Islampur	Sadar	Batiaghata	Jalma	Sadar	Ishwaripur
Village	Bamiya	Khalisha	Kismat Fultala	Krishnanagar	NA	Srifolkati
DecLat	22.4508	22.6195	22.7251	22.7102	22.5845	22.2926
DecLon	89.2833	89.5086	89.5216	89.7583	89.3124	89.1202
StnName	Koyra	Dakop	Kismat_Fultola	Krishnanagar	Paikgachha	Shyamnagar
StnType	Soil water	Soil water	Soil water	Soil water	Soil water	Soil water
ECAvgMin	1000	800	600	900	1300	1000

ECAvgMax	12500	14200	27300	15600	39300	12500
ECAvgLTMed	3808	4074	3212	2600	6250	3885
ECAvgLTavg	4544	4669	4717	4010	8060	4564
ECAvgLTsd	2398	2620	4256	2880	5920	2384
ECAvgDRYavg	5421	5746	5873	5017	10089	5447
ECAvgWETavg	3316	3163	3099	2600	5219	3327
ECavgLinTD	-62.70	-78.09	-126.35	-27.40	-236.07	-61.87
ECavgLNTDerr	28.71	31.28	50.82	34.80	69.90	28.55
ECavgLNTDp	0.03	0.01	0.01	0.43	0.00	0.03
ECavgSenTD	-79.04	-100.74	-45.45	-30.88	-200.00	-76.64
ECavgSenTDp	0.00	0.00	0.03	0.10	0.00	0.00
ECavgSSenTD	-55.29	-80.52	-27.92	-7.00	-185.71	-51.68
ECavgSTLTD	0.57	4.05	-80.09	-8.81	-16.27	2.67
ECavgMKTau	-0.15	-0.15	-0.10	-0.07	-0.17	-0.14
ECavgSMKTau	-0.24	-0.25	-0.10	-0.04	-0.26	-0.21
ECavgDryTD	-35.95	-31.52	-114.98	19.63	-158.65	-34.31
ECavgDryTDerr	38.50	40.53	74.32	48.12	97.49	37.97
ECavgDryTDp	0.35	0.44	0.12	0.68	0.11	0.37
ECavgWetTD	-91.72	-132.97	-131.13	-83.53	-325.08	-91.96
ECavgWetTDerr	30.86	32.06	50.87	34.88	68.76	31.03
ECavgWetTDp	0.00	0.00	0.01	0.02	0.00	0.00
ECavgSTLt (%)	7.45	9.92	10.86	15.60	5.12	7.46
ECavgSTLs (%)	81.90	77.21	48.12	46.93	61.37	81.43
ECavgSTLr (%)	10.65	12.87	41.02	37.48	33.51	11.10

Explanation: EC=electrical conductivity; all values are in µS/cm, but for trend it's µS/cm/year

ECAvgMin = Long-term average minimum salinity
ECAvgMax = Long-term average maximum salinity
ECAvgLTMed = Long-term average median salinity
ECAvgLTavg = Long-term average salinity
ECAvgLTsd = Long-term standard deviation salinity
ECAvgDRYavg = Dry-season average salinity
ECAvgWETavg = Wet-season average salinity
ECavgLinTD = Long-term linear trend in salinity
ECavgLNTDerr = Long-term trend standard error
ECavgLNTDp = Long-term trend statistical significance or *p* value
ECavgSenTD = Long-term Sen's slope or trend
ECavgSenTDp = Long-term Sen's trend statistical significance or *p* value
ECavgSSenTD = Long-term seasonal Sen's slope or trend
ECavgSTLTD = Long-term STL trend
ECavgMKTau = Mann-Kendall Tau value (strength and direction of trends)
ECavgSMKTau = Seasonal Mann-Kendall Tau value (strength and direction of trends)
ECavgDryTD = Long-term dry-season trend
ECavgDryTDerr = Long-term dry-season trend standard error
ECavgDryTDp = Long-term dry-season trend statistical significance or *p* value
ECavgWetTD = Long-term wet-season trend
ECavgWetTDerr = Long-term wet-season trend standard error
ECavgWetTDp = Long-term wet-season trend statistical significance or *p* value
ECavgSTLt = STL decomposed trend component explaining the variance (percent)
ECavgSTLs = STL decomposed seasonal component explaining the variance (percent)
ECavgSTLr = STL decomposed residual component explaining the variance (percent)

Table S3. Descriptive statistics, trends and seasonal-trend decomposition of river water salinity data. There are 13 river water salinity monitoring stations in southwestern coastal Bangladesh.

StationName	Daratana	Madhumati	Panguchhi	Poshur	Bhadra	Kazibachha	Shailmari
StationID	R12	R11	R13	R10	R6	R8	R7
Parameter	River water	River water	River water	River water	River water	River water	River water
District	Bagerhat	Bagerhat	Bagerhat	Bagerhat	Khulna	Khulna	Khulna
Upazila	Sadar	Mollahat	Morrelganj	Mongla	Dumuria	Batiaghata	Dumuria
Union	Sadar	Udaypur	Sadar	Sadar	Khornia	Batiaghata	Gutudia
Village	Daratana	Ferryghat	NA	NA	Khornia	Fultala	Koiya
DecLat	22.6439	22.9290	22.4663	22.4728	22.8226	22.6933	22.7893
DecLon	89.8036	89.8109	89.8631	89.6017	89.3533	89.5328	89.4852
StnName	Daratana	Madhumati	Panguchhi	Poshur	Bhadra	Kazibachha	Shailmari
StnType	River water	River water	River water	River water	River water	River water	River water
ECAvgMin	480	900	300	300	300	200	200
ECAvgMax	24900	18600	26100	33700	30700	33200	33000
ECAvgLTMed	4050	2400	2850	4600	3876	1999	1950
ECAvgLTavg	6808	4097	4617	8881	7878	7489	6206
ECAvgLTsd	6209	3525	4440	9473	7795	9201	7483
ECAvgDRYavg	8475	5287	6143	12480	9320	11196	8637
ECAvgWETavg	4474	2431	2480	3841	5859	2300	2803
ECavgLinTD	-182.44	-111.21	-147.21	-91.62	-84.83	-22.78	99.69
ECavgLNTDerr	74.15	42.00	52.83	114.47	94.16	111.33	90.31
ECavgLNTDp	0.01	0.01	0.01	0.42	0.37	0.84	0.27
ECavgSenTD	-107.59	-45.28	-80.00	-24.87	-26.09	0.00	12.44
ECavgSenTDp	0.01	0.02	0.01	0.35	0.50	0.57	0.58
ECavgSSenTD	-100.00	-30.00	-75.00	-10.00	17.16	9.09	28.57
ECavgSTLTD	-53.54	-100.51	-166.49	-4.41	24.82	-32.32	74.76
ECavgMKTau	-0.12	-0.10	-0.13	-0.04	-0.03	0.03	0.03
ECavgSMKTau	-0.22	-0.13	-0.20	-0.05	0.04	0.11	0.08
ECavgDryTD	-156.35	-86.77	-149.27	-84.72	25.73	-45.96	203.90
ECavgDryTDerr	107.25	58.68	76.33	153.15	126.67	152.34	128.58
ECavgDryTDp	0.15	0.14	0.05	0.58	0.84	0.76	0.12
ECavgWetTD	-203.09	-133.93	-129.46	-66.06	-226.37	46.16	-22.85
ECavgWetTDerr	78.04	42.55	45.00	121.10	131.17	99.81	86.42
ECavgWetTDp	0.01	0.00	0.00	0.59	0.09	0.64	0.79
ECavgSTLt (%)	2.73	12.45	5.56	0.51	1.27	0.77	1.62
ECavgSTLs (%)	79.18	51.19	66.30	84.37	78.98	84.55	75.96
ECavgSTLr (%)	18.09	36.36	28.15	15.12	19.74	14.68	22.42

StationName	Shailmari	Shibsha	Rupsha	Kakshiali	Betna	Kapotaksha	Morichap
StationID	R7	R5	R9	R1	R3	R4	R2
Parameter	River water	River water	River water	River water	River water	River water	River water
District	Khulna	Khulna	Khulna	Satkhira	Satkhira	Satkhira	Satkhira
Upazila	Dumuria	Paikgachha	Sadar	Kaliganj	Sadar	Tala	Ashashuni
Union	Gutudia	Sadar	NA	Sadar	Labsa	Kumira	Sadar
Village	Koiya	NA	Ferryghat	Kaliganj	Benarpota	Patkelghata	Ashashuni
DecLat	22.7893	22.5844	22.7770	22.4580	22.7506	22.7654	22.5497
DecLon	89.4852	89.3131	89.5867	89.0386	89.1065	89.1718	89.1756

StnName	Shailmari	Shibsha	Rupsha	Kakshiali	Betna	Kapotaksha	Morichap
StnType	River water	River water	River water	River water	River water	River water	River water
ECAvgMin	200	500	200	400	300	300	400
ECAvgMax	33000	33600	30200	36600	33000	26400	35500
ECAvgLTMed	1950	7258	800	9000	3700	1972	8872
ECAvgLTavg	6206	11297	5709	12350	7888	5016	12218
ECAvgLTsd	7483	9685	7914	9866	8381	6062	9863
ECAvgDRYavg	8637	13716	8632	14803	10050	6341	14576
ECAvgWETavg	2803	7909	1617	8917	4861	3160	8917
ECavgLinTD	99.69	-94.94	-43.31	-99.85	-208.71	-195.91	16.05
ECavgLNTDerr	90.31	117.03	95.73	119.20	100.47	72.19	119.35
ECavgLNTDp	0.27	0.42	0.65	0.40	0.04	0.01	0.89
ECavgSenTD	12.44	-55.94	0.00	-50.88	-69.57	-15.60	30.49
ECavgSenTDp	0.58	0.37	0.69	0.55	0.09	0.43	0.68
ECavgSSenTD	28.57	-10.00	0.00	0.00	-42.86	-9.93	92.58
ECavgSTLTD	74.76	78.81	-5.72	-42.87	-100.58	-204.90	214.87
ECavgMKTau	0.03	-0.04	0.02	-0.03	-0.08	-0.04	0.02
ECavgSMKTau	0.08	-0.02	0.08	0.00	-0.08	-0.04	0.12
ECavgDryTD	203.90	-16.93	-69.13	-11.91	-169.08	-142.62	102.49
ECavgDryTDerr	128.58	155.69	136.51	159.21	136.21	101.04	159.66
ECavgDryTDp	0.12	0.91	0.61	0.94	0.22	0.16	0.52
ECavgWetTD	-22.85	-181.15	21.54	-199.63	-243.54	-258.08	-82.50
ECavgWetTDerr	86.42	157.91	79.89	159.69	129.33	90.09	161.00
ECavgWetTDp	0.79	0.25	0.79	0.21	0.06	0.01	0.61
ECavgSTLt	1.62	0.60	0.89	0.63	2.91	4.63	0.68
ECavgSTLs	75.96	86.10	82.38	85.98	73.60	70.64	86.69
ECavgSTLr	22.42	13.30	16.73	13.39	23.48	24.74	12.63

Note: for explanation of the statistics, see Table S1.

Table S4. Summary statistics of multiple linear regression (MLR) models for soil salinity in southwestern coastal Bangladesh. Model is fitted with the original data frame with missing observations in number of covariates.

	**log(AvgSoil)**			
Predictors	*Estimates*	*CI*	*Statistic*	*p*
(Intercept)	6.76261 ***	3.75584 – 9.76937	4.49315	**<0.001**
AvgRain	-0.00028	-0.00248 – 0.00192	-0.25418	0.800
AvgET	-0.00918	-0.01939 – 0.00104	-1.79350	0.078
AvgTemp	0.13163 ***	0.06182 – 0.20145	3.76653	**<0.001**
AvgSM	-0.00016	-0.00505 – 0.00472	-0.06687	0.947
AvgRO	-0.00063	-0.00892 – 0.00766	-0.15175	0.880
SLanom	-0.00150 **	-0.00260 – -0.00040	-2.72954	**0.008**
Ssalinity	0.00001	-0.00003 – 0.00005	0.56218	0.576
RivDisch	-0.00004	-0.00010 – 0.00003	-1.15491	0.252
Cyclone	0.17960	-0.37907 – 0.73827	0.64223	0.523
SWLm	0.89004 ***	0.50333 – 1.27675	4.59792	**<0.001**
GWLbgl	0.23307	-0.13305 – 0.59919	1.27175	0.208
NDVI	-2.96468 ***	-4.39550 – -1.53386	-4.13931	**<0.001**
Observations	77 out of 222 due to missingness			
R^2 / R^2 adjusted	0.78 / 0.73			
AIC	1398.49			
log-Likelihood	-19.33			
				* p<0.05 ** p<0.01 *** p<0.001

Covariates or predictors used in these models are: AvgRain=average rainfall (mm); AvgTemp=average temperature (°C); SLanom=sea level anomaly (m); Ssalinity=sea surface salinity (µS/cm); RivDisch=river discharge (); SWLm=surface water levels (m); Cyclone=occurrence of tropical cyclones (binary); GWLbgl=groundwater level as depth below ground level (m, bgl) expressed as negative values to indicate depth; NDVI=Normalised Difference Vegetation Index; RivDisch:SWLm=an interaction term between river discharge (RivDisch) and surface water levels (SWLm).

Table S5. Summary statistics of multiple linear regression (MLR) models for soil salinity in southwestern coastal Bangladesh. Model is fitted with the data frame where missing observations in the covariates were imputed using random forest algorithm.

	**log(AvgSoil)**			
Predictors	*Estimates*	*CI*	*Statistic*	*p*
(Intercept)	6.09269 ***	5.29841 – 6.88698	15.12097	**<0.001**
AvgRain	-0.00034	-0.00082 – 0.00013	-1.41704	0.158
AvgTemp	0.06172 ***	0.03518 – 0.08827	4.58301	**<0.001**
SLanom	-0.00179 ***	-0.00240 – -0.00118	-5.77627	**<0.001**
Ssalinity	0.00005 ***	0.00003 – 0.00007	5.01821	**<0.001**
RivDisch	-0.00007	-0.00015 – 0.00001	-1.69756	0.091
SWLm	0.50218 **	0.19986 – 0.80450	3.27443	**0.001**
Cyclone	0.24994 *	0.03551 – 0.46437	2.29773	**0.023**
GWLbgl	0.20907	-0.00683 – 0.42496	1.90892	0.058
NDVI	-1.81439 ***	-2.87438 – -0.75440	-3.37424	**0.001**
RivDisch × SWLm	0.00003	-0.00002 – 0.00008	1.34166	0.181
Observations	222 following imputation of missing values			
R^2 / R^2 adjusted	0.72 / 0.70			
AIC	3961.47			
log-Likelihood	-90.81			
** p<0.05 ** p<0.01 *** p<0.001*				

Covariates or predictors used in these models are: AvgRain=average rainfall (mm); AvgTemp=average temperature (°C); SLanom=sea level anomaly (m); Ssalinity=sea surface salinity (μS/cm); RivDisch=river discharge (); SWLm=surface water levels (m); Cyclone=occurrence of tropical cyclones (binary); GWLbgl=groundwater level as depth below ground level (m, bgl) expressed as negative values to indicate depth; NDVI=Normalised Difference Vegetation Index; RivDisch:SWLm=an interaction term between river discharge (RivDisch) and surface water levels (SWLm).

Table S6. Summary statistics of multiple linear regression (MLR) models for river water salinity in southwestern coastal Bangladesh. Model is fitted with the original data frame with missing observations in number of covariates.

	**log(AvgRiv)**			
Predictors	*Estimates*	*CI*	*Statistic*	*p*
(Intercept)	2.68895	-0.08346 – 5.46137	1.93759	0.057
AvgRain	-0.00044	-0.00247 – 0.00159	-0.43367	0.666
AvgET	-0.00732	-0.01674 – 0.00210	-1.55192	0.126
AvgTemp	0.10807 **	0.04370 – 0.17245	3.35379	**0.001**
AvgSM	0.00117	-0.00334 – 0.00567	0.51717	0.607
AvgRO	-0.00276	-0.01040 – 0.00488	-0.72123	0.473
SLanom	0.00032	-0.00070 – 0.00133	0.62090	0.537
Ssalinity	0.00008 ***	0.00004 – 0.00012	4.24912	**<0.001**
SWLm	-0.06926	-0.42583 – 0.28731	-0.38805	0.699
RivDisch	-0.00000	-0.00006 – 0.00005	-0.08220	0.935
Cyclone	0.18884	-0.32629 – 0.70397	0.73234	0.467
GWLbgl	-0.25259	-0.59018 – 0.08499	-1.49476	0.140
NDVI	-0.09180	-1.41110 – 1.22750	-0.13901	0.890
Observations	77 out of 222 due to missingness			
R^2 / R^2 adjusted	0.92 / 0.91			
AIC	1365.79			
log-Likelihood	-13.09			
				* p<0.05 ** p<0.01 *** p<0.001

Covariates or predictors used in these models are: AvgRain=average rainfall (mm); AvgTemp=average temperature (°C); SLanom=sea level anomaly (m); Ssalinity=sea surface salinity (µS/cm); RivDisch=river discharge (); SWLm=surface water levels (m); Cyclone=occurrence of tropical cyclones (binary); GWLbgl=groundwater level as depth below ground level (m, bgl) expressed as negative values to indicate depth; NDVI=Normalised Difference Vegetation Index; RivDisch:SWLm=an interaction term between river discharge (RivDisch) and surface water levels (SWLm).

Table S7. Summary statistics of multiple linear regression (MLR) models for river water salinity in southwestern coastal Bangladesh. Model is fitted with the data frame where missing observations in the covariates were imputed using random forest algorithm.

	**log(AvgRiv)**			
Predictors	*Estimates*	*CI*	*Statistic*	*p*
(Intercept)	4.05117 ***	3.30489 – 4.79746	10.70093	**<0.001**
AvgRain	0.00042	-0.00003 – 0.00087	1.86026	0.064
AvgTemp	0.06078 ***	0.03584 – 0.08573	4.80319	**<0.001**
SLanom	-0.00061 *	-0.00118 – -0.00004	-2.09468	**0.037**
Ssalinity	0.00007 ***	0.00005 – 0.00009	7.62605	**<0.001**
RivDisch	-0.00009 *	-0.00016 – -0.00002	-2.39365	**0.018**
SWLm	-0.14517	-0.42922 – 0.13889	-1.00743	0.315
Cyclone	0.03538	-0.16610 – 0.23685	0.34612	0.730
GWLbgl	-0.35426 ***	-0.55711 – -0.15141	-3.44268	**0.001**
NDVI	-0.29875	-1.29468 – 0.69718	-0.59132	0.555
RivDisch × SWLm	0.00005 *	0.00000 – 0.00009	2.13027	**0.034**
Observations	222 following imputation of missing values			
R^2 / R^2 adjusted	0.89 / 0.89			
AIC	3932.91			
log-Likelihood	-76.98			
* *p<0.05* ** *p<0.01* *** *p<0.001*				

Covariates or predictors used in these models are: AvgRain=average rainfall (mm); AvgTemp=average temperature (°C); SLanom=sea level anomaly (m); Ssalinity=sea surface salinity (µS/cm); RivDisch=river discharge (); SWLm=surface water levels (m); Cyclone=occurrence of tropical cyclones (binary); GWLbgl=groundwater level as depth below ground level (m, bgl) expressed as negative values to indicate depth; NDVI=Normalised Difference Vegetation Index; RivDisch:SWLm=an interaction term between river discharge (RivDisch) and surface water levels (SWLm).

Table S8. Summary statistics of multiple linear regression (MLR) models for soil salinity in southwestern coastal Bangladesh. There are four models here: M1=modelling soil salinity with only meteorological factors; M2=model with climatological factors; M3=model with hydrological and hydrogeological factors; and M4=land-use/land-cover and anthropogenic factors.

	**M1: log(AvgSoil)**		**M2: log(AvgSoil)**		**M3: log(AvgSoil)**		**M4:log(AvgSoil)**	
Predictors	*Estimates*	*p*	*Estimates*	*p*	*Estimates*	*p*	*Estimates*	*p*
(Intercept)	6.91764 ***	**<0.001**	6.47509 ***	**<0.001**	9.01788 ***	**<0.001**	8.08638 ***	**<0.001**
**Meteorological variables:**								
AvgRain	0.00005	0.864						
AvgTemp	0.12383 ***	**<0.001**						
AvgET	-0.01831 ***	**<0.001**						
Cyclone	0.36345 **	**0.008**						
**Climatological factors:**								
SLanom			-0.00101 ***	**<0.001**				
Ssalinity			0.00006 ***	**<0.001**				
**Hydrological / hydrogeological factors:**								
AvgSM					-0.00516 ***	**<0.001**		
AvgRO					0.00086	0.207		
RivDisch					-0.00001	0.115		
SWLm					0.58050 ***	**<0.001**		
**Land-use/Land-cover and anthropogenic factors:**								
†GWLbgl					-0.26329 **	**0.002**	-0.52758 ***	**<0.001**
NDVI							-1.34863 **	**0.001**
Observations	222		222		222		222	
R^2 / R^2 adjusted	0.513 / 0.504		0.623 / 0.620		0.624 / 0.615		0.572 / 0.568	
AIC	4070.002		4008.802		4014.770		4037.173	
log-Likelihood	-151.080		-122.480		-122.464		-136.665	
** p<0.05 ** p<0.01 *** p<0.001*								

Covariates or predictors used in these models are: AvgRain=average rainfall (mm); AvgTemp=average temperature (°C); SLanom=sea level anomaly (m); Ssalinity=sea surface salinity (µS/cm); RivDisch=river discharge (); SWLm=surface water levels (m); Cyclone=occurrence of tropical cyclones (binary); GWLbgl=groundwater level as depth below ground level (m, bgl) expressed as negative values to indicate depth; NDVI=Normalised Difference Vegetation Index. †Groundwater level is grouped under the land-use / land-cover and anthropogenic factor in this model though it is originally grouped under the hydrological / hydrogeological factors in this study.

Table S9. Summary statistics of multiple linear regression (MLR) models for river water salinity in southwestern coastal Bangladesh. There are four models here: M1=modelling soil salinity with only meteorological factors; M2=model with climatological factors; M3=model with hydrological and hydrogeological factors; and M4=land-use/land-cover and anthropogenic factors.

	**M1: log(AvgRiv)**		**M2: log(AvgRiv)**		**M3: log(AvgRiv)**		**M4: log(AvgRiv)**	
Predictors	*Estimates*	*p*	*Estimates*	*p*	*Estimates*	*p*	*Estimates*	*p*
(Intercept)	4.99027 ***	**<0.001**	3.95721 ***	**<0.001**	6.22472 ***	**<0.001**	5.63930 ***	**<0.001**
**Meteorological variables:**								
AvgRain	0.00040	0.303						
AvgTemp	0.23839 ***	**<0.001**						
AvgET	-0.03036 ***	**<0.001**						
Cyclone	0.45974 *	**0.016**						
**Climatological factors:**								
SLanom			0.00033	0.206				
Ssalinity			0.00013 ***	**<0.001**				
**Hydrological / hydrogeological factors:**								
AvgSM					-0.00137	0.204		
AvgRO					0.00174 *	**0.011**		
RivDisch					-0.00001	0.499		
SWLm					0.28066 *	**0.036**		
**Land-use/Land-cover and anthropogenic factors:**								
†GWLbgl					-1.13997 ***	**<0.001**	-1.22120 ***	**<0.001**
NDVI							0.64636	0.104
Observations	222		222		222		222	
R^2 / R^2 adjusted	0.597 / 0.589		0.830 / 0.828		0.839 / 0.835		0.830 / 0.828	
AIC	4217.287		4021.559		4015.525		4021.581	
log-Likelihood	-225.166		-129.302		-123.285		-129.313	
								** p<0.05 ** p<0.01 *** p<0.001*

Covariates or predictors used in these models are: AvgRain=average rainfall (mm); AvgTemp=average temperature (°C); SLanom=sea level anomaly (m); Ssalinity=sea surface salinity (µS/cm); RivDisch=river discharge (); SWLm=surface water levels (m); Cyclone=occurrence of tropical cyclones (binary); GWLbgl=groundwater level as depth below ground level (m, bgl) expressed as negative values to indicate depth; NDVI=Normalised Difference Vegetation Index. †Groundwater level is grouped under the land-use / land-cover and anthropogenic factor in this model though it is originally grouped under the hydrological / hydrogeological factors in this study.
